# Diffusion MRI with machine learning

**DOI:** 10.1162/imag_a_00353

**Published:** 2024-11-12

**Authors:** Davood Karimi, Simon K. Warfield

**Affiliations:** Department of Radiology, Boston Children’s Hospital and Harvard Medical School, Boston, MA, United States

**Keywords:** diffusion MRI, machine learning, artificial intelligence, deep learning

## Abstract

Diffusion-weighted magnetic resonance imaging (dMRI) of the brain offers unique capabilities including noninvasive probing of tissue microstructure and structural connectivity. It is widely used for clinical assessment of disease and injury, and for neuroscience research. Analyzing the dMRI data to extract useful information for medical and scientific purposes can be challenging. The dMRI measurements may suffer from strong noise and artifacts, and may exhibit high intersession and interscanner variability in the data, as well as intersubject heterogeneity in brain structure. Moreover, the relationship between measurements and the phenomena of interest can be highly complex. Recent years have witnessed increasing use of machine learning methods for dMRI analysis. This manuscript aims to assess these efforts, with a focus on methods that have addressed data preprocessing and harmonization, microstructure mapping, tractography, and white matter tract analysis. We study the main findings, strengths, and weaknesses of the existing methods and suggest topics for future research. We find that machine learning may be exceptionally suited to tackle some of the difficult tasks in dMRI analysis. However, for this to happen, several shortcomings of existing methods and critical unresolved issues need to be addressed. There is a pressing need to improve evaluation practices, to increase the availability of rich training datasets and validation benchmarks, as well as model generalizability, reliability, and explainability concerns.

## 1 Introduction

### 1.1 Background and motivation

Diffusion-weighted magnetic resonance imaging (dMRI) is a widely used medical imaging modality (Johansen-Berg & Behrens, 2013;Le Bihan, 2014). It has a unique role in neuroimaging, where it stands as the only noninvasive method for probing the tissue microstructural makeup and structural connectivity of the brain (Lerch et al., 2017;Tournier, 2019). It has facilitated the study of normal brain development and quantitative characterization of the impact of diseases and disorders (Bodini & Ciccarelli, 2009;Salat, 2014). As a result, dMRI has become an indispensable tool in medicine and neuroscience, and it has been a major component of large neuroimaging initiatives (Miller et al., 2016;Van Essen et al., 2013).

Raw dMRI data can suffer from a host of imperfections and artifacts (Jones, 2010;Tax et al., 2022). Yet, these data need to be analyzed to uncover subtle differences or minute changes that reflect the underlying normal or abnormal variations in brain development. These neurodevelopmental processes, in turn, are highly complex, heterogeneous, and multifactorial. Consequently, development and validation of computational methods for dMRI analysis are difficult. Accurate and reproducible processing of dMRI data has been a long-standing challenge, and thousands of research papers have been devoted to addressing its various aspects. Several computational pipelines and software projects have aimed at standardizing and streamlining some of the more routine dMRI computations (Garyfallidis et al., 2014;Theaud et al., 2020;Tournier et al., 2019). However, there are many persistent challenges that have not been fully addressed and there is an urgent need for new methods to enable higher accuracy, reproducibility, reliability, and computational speed (Bach et al., 2014;Jones & Cercignani, 2010;Tax et al., 2022).

Classical dMRI analysis methods have appropriately been based on conventional signal processing, biophysical modeling, and numerical optimization techniques. Meanwhile, many studies have advocated for machine learning and data-driven techniques (Golkov et al., 2016;Poulin et al., 2019;Wasserthal, Neher, & Maier-Hein, 2018) motivated by the opportunities to exploit advances in hardware, new software libraries, and models learned from data. These techniques have become more popular in recent years. This trend has been driven by more powerful machine learning models (mostly based on deep neural networks) and a greater appreciation of the power and flexibility of these methods. Overall, these methods have been shown to possess the potential to improve the speed, accuracy, and reproducibility of various computations in dMRI analysis such as data preprocessing (Hu et al., 2020) and harmonization (Moyer et al., 2020), tissue microstructure mapping (de Almeida Martins, Nilsson, Lampinen, Palombo, While, et al., 2021;Golkov et al., 2016;Karimi, Jaimes, et al., 2021), tractography (Poulin et al., 2019), tract-specific analysis (Wasserthal, Neher, & Maier-Hein, 2018), and population studies (B. Li, Niessen, Klein, de Groot, et al., 2021). It appears that recent studies herald a new generation of dMRI analysis methods that may significantly complement, if not at least in some cases entirely replace, the more conventional techniques. Therefore, it is good time to review, summarize, and critically assess the achievements of these studies, highlight their shortcomings and limitations, and point out future study that may contribute to this new field in dMRI.

### 1.2 Scope and organization of this manuscript

This manuscript focuses almost entirely on applications in neuroimaging. It deals with the processing steps after the reconstruction of the so-called q-space data. It is concerned with data preprocessing steps such as denoising and artifact correction as well as downstream computations such as white matter microstructure mapping and tract-specific analysis.

The search to find the relevant studies to be included in this paper was conducted in PubMed and Google Scholar. The initial search was performed in June 2023 and then repeated in June 2024. The search terms included “diffusion MRI” and “machine learning” or “deep learning.” More detailed searches were performed in reputable journals (e.g., Medical Image Analysis and IEEE Transactions on Medical Imaging) and conferences (e.g., MICCAI) that have a focus on this topic. Given the wide scope of work in this domain, we sought to focus on papers with interesting methodological advances or extensive experimental results.

**Fig. 1.**
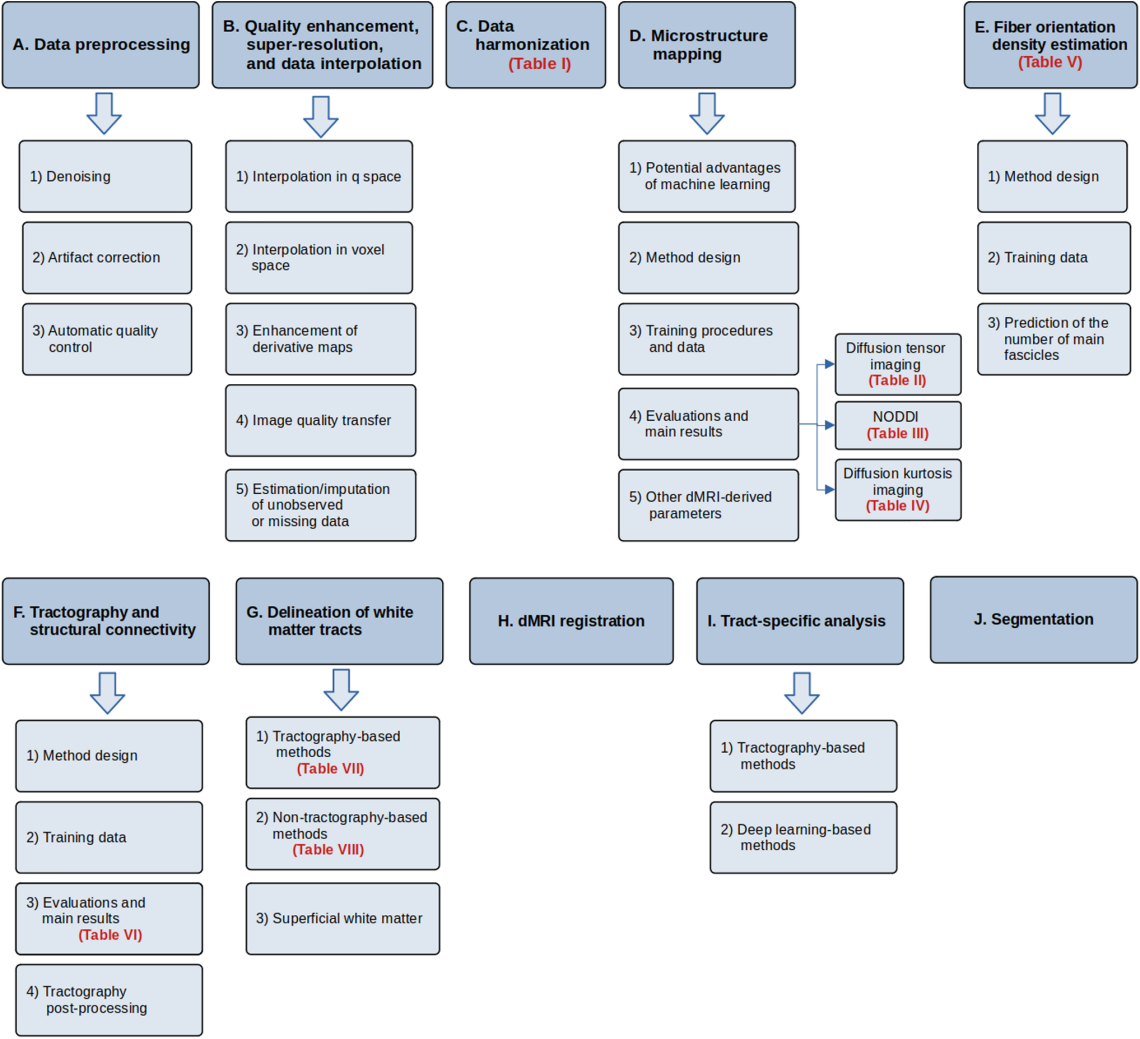
Outline of the 10 classes of methods that have been surveyed inSection 4.

**Fig. 2.**
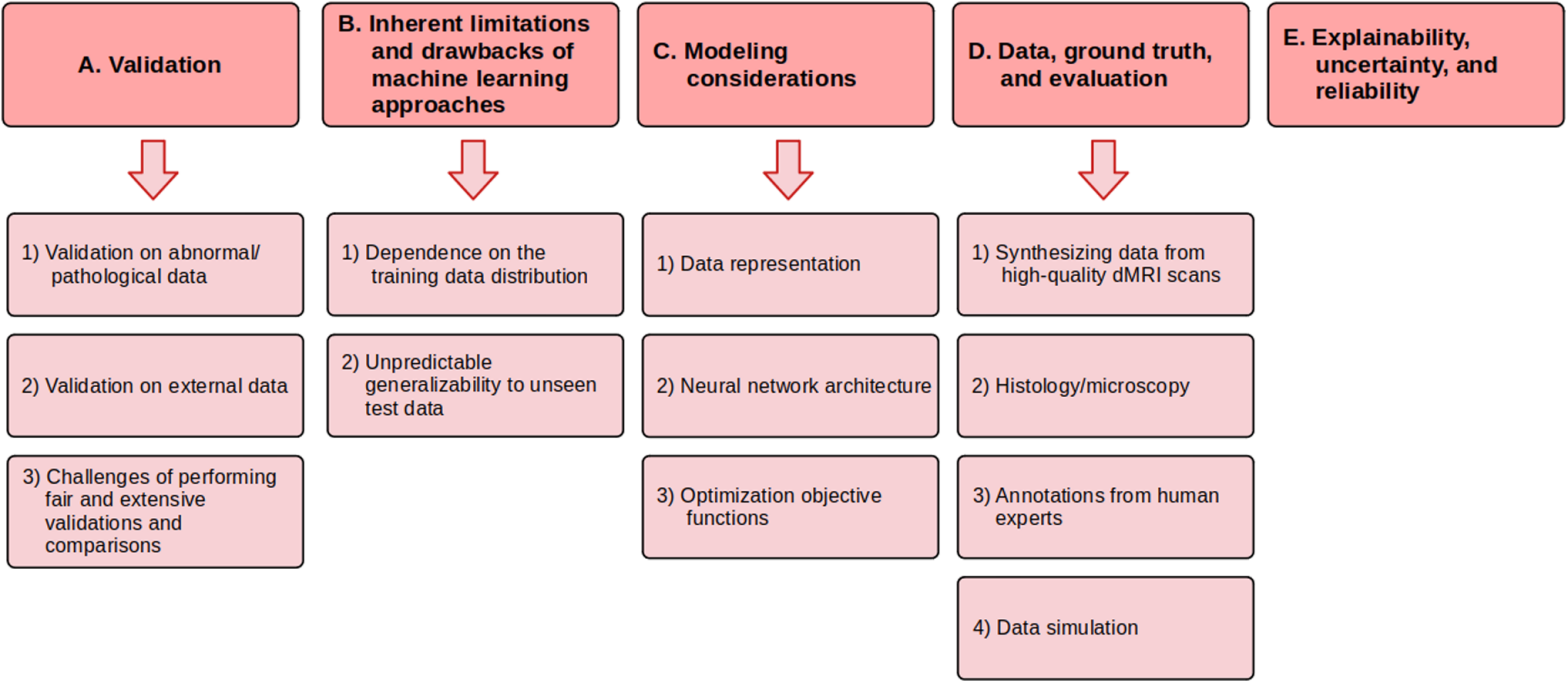
Outline of the main aspects of application of machine learning in dMRI that are discussed inSection 5.

This paper starts with a brief description of challenges in dMRI data analysis (Section 2) and general overview of the reasons why machine learning may be effective in addressing these challenges (Section 3).Section 4reviews prior studies that have used machine learning in dMRI. The main applications considered include data preprocessing and quality enhancement, data harmonization, estimation of tissue microstructure and fiber orientation, tractography, tract-specific analysis, registration, and segmentation.Figure 1shows the outline of that section. Following that,Section 5discusses the main technical considerations, challenges, and open questions. Some of the main topics discussed in that section include validation approaches, inherent limitations of machine learning, data, ground truth, and modeling considerations, as well as model explainability, uncertainty, and reliability concerns.Figure 2shows the outline of that section. A shortSection 6will present the closing remarks.

## 2 The Challenging Nature of DMRI Analysis

The challenges and pitfalls of analyzing the dMRI data have been discussed in dedicated publications (Jones & Cercignani, 2010;Jones et al., 2013;Tax et al., 2022). A primary source of difficulty in dMRI analysis is measurement noise and artifacts (Pierpaoli, 2010;Tax et al., 2022). Measurement noise can significantly alter the analysis results even in relatively simple computations such as diffusion tensor estimation (Jones & Basser, 2004;Roalf et al., 2016). It can be difficult to suppress the noise and artifacts due to their complex distributions. The echo planar imaging method that is used to acquire dMRI measurements can give rise to eddy current- and susceptibility-induced distortions. Subject motion during data acquisition is another persistent challenge in MRI. These factors can significantly impact the accuracy of quantitative tissue microstructure mapping and structural connectivity analysis (Baum et al., 2018;Oldham et al., 2020;Yendiki et al., 2014). Furthermore, the relationship between the dMRI measurements and the underlying microstructure of neuronal tissue is complex (Novikov et al., 2018,^2019^). Another source of difficulty is the measurement requirements of advanced dMRI models. Estimation of multicompartment models of tissue microstructure and of complex white matter fiber configurations requires densely sampled q-space data. Models often seek to represent properties of brain tissue with parameters corresponding to aspects of microstructure. Classical signal processing approaches are concerned with characterizing the identifiability of model parameters, and with characterizing any potential bias or variance in estimated values of parameters. For some model parameters to be identifiable, imaging strategies may need to be carefully chosen to obtain suitable measurements. Obtaining such measurements may require long scan times, leading to a trade-off between the acquisition duration and the phenomena captured by a model. Even with a reasonably large number of measurements, standard computational methods can produce erroneous results (Kerkelä, Seunarine, Henriques, et al., 2022). As only one example, conventional linear and nonlinear least-squares techniques for diffusion kurtosis imaging (DKI;Jensen et al., 2005) tend to produce physically implausible results (Neto Henriques, 2018;Tabesh et al., 2011). Streamline tractography and tract-specific analysis face inherent challenges and ambiguities such as fiber crossings and bottlenecks (Calixto, Soldatelli, et al., 2024;Jones et al., 2013;Maier-Hein et al., 2017;Thomas et al., 2014), which can give rise to high false positive rates and erroneous results. Cross-subject comparisons and population studies can also be significantly hampered by high interscanner data heterogeneity (Cai et al., 2021a;Ning et al., 2020). Furthermore, most applications are also challenged by paucity of ground truth and lack of universally accepted performance metrics.

## 3 Why Machine Learning May Help

Machine learning encompasses a rich set of flexible and powerful methods that have the potential to improve upon the more conventional techniques for dMRI analysis. The advantages of machine learning methods over classical techniques depend on the specific tasks, which are discussed in more detail in the following sections. Here, we list some of the main reasons why machine learning may be useful for dMRI analysis.

- Machine learning methods can learn the complex relationships between the input and output from observing a large number of examples. This may be a fundamentally more plausible and more powerful approach than conventional methods that are based on approximate biophysical models (Fick et al., 2017) or ad-hoc and simplistic rules such as those used in tractography (Poulin et al., 2019).
- Machine learning models can represent highly complex functions. For neural networks, universal approximation theorems state that they can represent all functions of practical interest (Hornik et al., 1989). This means that machine learning models are far less restricted than conventional methods in terms of the complexity of the phenomena that they can represent. This can be a significant advantage because the true mapping between the input and output may be outside the scope of the mathematical models imposed by conventional methods (Nedjati-Gilani et al., 2014,^2017^).
- As a result of the above properties, machine learning methods can simplify the complex multistage pipelines into simpler end-to-end methods. Many dMRI analysis tasks such as estimation of tissue microstructure or segmentation of white matter tracts rely on a sequence of computations that are optimized separately and independently (Golkov et al., 2016;Wasserthal, Neher, & Maier-Hein, 2018). With machine learning methods, it is often possible to combine these into a single model that can be optimized jointly (Wasserthal, Neher, & Maier-Hein, 2018).
- Many machine learning models can effectively and seamlessly integrate various inputs, constraints, and sources of prior knowledge, such as other MRI contrasts or spatial information. As an example, recent studies have shown that deep learning methods can easily leverage anatomical MRI data (on top of dMRI data) to improve microstructure estimation (Tian et al., 2020), super-resolution (Qin, Li, et al., 2021), tissue segmentation (Golkov et al., 2016), and distortion correction (Hu et al., 2020). Given the rich spatial regularity in many neuroimaging problems, incorporation of spatial information may significantly improve the model performance. Spatial information can be too complex to mathematically formulate with conventional methods and, if not done properly, can have a negative impact on performance (Gruen et al., 2023). Machine learning models, in contrast, can effortlessly learn this information directly from data. Modern neural networks such as Convolutional Neural Networks (CNNs), graph CNNs, and transformers have been shown to be especially well suited for this purpose (Hong, Kim, et al., 2019;Karimi & Gholipour, 2022b;Tian et al., 2020;Weine et al., 2022).
- Machine learning methods may offer much faster computation (Kaandorp et al., 2021). This is especially the case for neural networks, where the models consist of a large number of basic operations that can be parallelized on graphical processing units (GPUs). Although the training of these models may require much time, prediction/inference on a new data sample can be orders of magnitude faster than conventional methods (Barbieri et al., 2020;de Almeida Martins, Nilsson, Lampinen, Palombo, While, et al., 2021;Kerkelä, Seunarine, Henriques, et al., 2022;Wasserthal, Neher, & Maier-Hein, 2018). The speed advantage has become increasingly more important as image resolution and dataset size continue to grow.
- Machine learning models may be trained on data with various types and different levels of imperfections such as noise, motion, suboptimal acquisition protocols, and imaging artifacts. This way, they may gain a degree of robustness with respect to these data imperfections by learning to factor them out in their computations (de Almeida Martins, Nilsson, Lampinen, Palombo, While, et al., 2021;Gong et al., 2019,^2021^;H. Li et al., 2021;Neher et al., 2015;Weine et al., 2022). This can be a unique advantage since mathematically modeling these data imperfections can be difficult or impossible. It has been reported, for example byWegmayr et al. (2018)for tractography, that machine learning models trained on data with noisy labels may produce more accurate and less noisy predictions on test data.Weine et al. (2022)developed a pipeline for generating synthetic cardiac diffusion tensor imaging (DTI) data that incorporated simulated heart motion and showed that a deep learning model trained on such data outperformed standard DTI estimation methods.
- Because machine learning methods are model free, they avoid the simplifying approximations, such as Gaussianity of the diffusion process or Ricianity of the measurement noise, which are bound to impact the performance of conventional methods. Moreover, existing mathematical models may be intractable and standard computational methods may generate unreliable results (Hill et al., 2021;Nedjati-Gilani et al., 2017). If adequate training data are available, machine learning models can side step these stumbling blocks and learn the underlying mapping from data.
- By offering new ways of analyzing the dMRI data, machine learning methods provide new insights into the potential and limitations of this imaging modality. A good case in point is the recent study ofCai, Lee, Newlin, Kerley, et al. (2023)on tractography, where the authors have investigated the possibility of performing tractography based purely on anatomical MRI data without any diffusion encoding. Their results suggest that tractography and structural connectivity analysis based on anatomical MRI may be comparable with those based on dMRI measurements, raising questions about whether standard streamline tractography is driven by tissue microstructure. Other studies have used autoencoders and similar bottleneck networks to characterize the information content of dMRI signal and discover common features between different biophysical models (Nath et al., 2021;Zucchelli et al., 2021a).

## 4 Prior Studies on DMRI Analysis with Machine Learning

Due to its unique advantages mentioned above, machine learning has the potential to improve the accuracy, robustness, generalizability, and computational speed for many dMRI analysis tasks. This section describes some of the recent studies that have investigated this potential. The diagram inFigure 1shows the outline of the methods covered in this section.

### 4.1 Data preprocessing

Diffusion-weighted MRI measurements suffer from artifacts and imperfections that can influence the results of downstream analysis steps and the conclusions drawn from the data. Therefore, proper data preprocessing is an essential first step for dMRI analysis (Tax et al., 2022). This section describes the recent progress in using machine learning methods to improve preprocessing of dMRI data.

#### 4.1.1 Denoising

Measurement noise in dMRI has a spatially varying distribution and depends on factors such as acquisition and reconstruction methods. It is typically approximated using Rician or noncentral chi square distributions (Canales-Rodrıguez et al., 2015;Dietrich et al., 2008). The noise is stronger at high b-values that are essential for estimating fiber orientation and multicompartment models, and it is of high practical importance because it can influence the analysis results in subtle but significant and complex ways (Jones, 2004;Jones & Basser, 2004;Roalf et al., 2016). Denoising methods based on the principal components analysis and nonlocal means have been popular in dMRI (Manjón et al., 2010;Veraart et al., 2016). These methods were often adapted to the dMRI setting in innovative ways. For example,St-Jean et al. (2016)combined the nonlocal matching with dictionary learning. In order to adapt the nonlocal means method to curved non-Euclidean domain of dMRI signals,Chen et al. (2017)developed a method based on convolutions on graphs that enabled computing multiscale features from dMRI data.

In recent years, several neural network-based image denoising algorithms have been proposed by the computer vision society. These methods, such as Noise2Noise (Lehtinen et al., 2018), Noise2Void (Krull et al., 2019), and Noise2Self (Batson & Royer, 2019), have often surpassed the performance of classical denoising algorithms. They offer several advantages that are especially useful for medical imaging applications including dMRI: (1) They do not need to model the image prior, (2) they do not need to know the noise distribution, and (3) they do not require clean images or paired noisy-clean images for training. The ideas presented in these methods have been adopted to develop dMRI denoising techniques in a few recent studies (Fadnavis et al., 2020;Tian et al., 2022).Fadnavis et al. (2020)proposed Patch2Self, which is based on learning locally linear relationships between different dMRI volumes. Patch2Self predicts the signal in one of the q-space elements (i.e., one channel in a 4D representation of the dMRI data) based on the other elements. In order to exploit spatial correlations, the model uses 3D spatial patches as input and target. Effectiveness of Patch2Self was demonstrated in a study on spinal cord (K. G. Schilling et al., 2021b), where Patch2Self improved FA repeatability and multiple sclerosis (MS) lesion conspicuity. SDnDTI (Tian et al., 2022) is another self-supervised method that uses a CNN model. It is specially tailored to single-shell data for DTI estimation. Given a set of N dMRI volumes (each acquired with a different gradient direction but the same gradient strength), SDnDTI selects subsets of six volumes and denoises them. This is done by training the CNN to take these six volumes as input and predict a low-noise target that is computed from all N volumes. This is repeated until all N volumes are denoised. Experiments show that SDnDTI is superior to standard methods such as those based on PCA and nonlocal means.

#### 4.1.2 Artifact correction

Diffusion MRI measurements can also suffer from various artifacts such as those caused by off-resonance fields, subject motion, and Gibbs ringing (Andersson, 2021;Pierpaoli, 2010). In the past two decades, much research has been devoted to computational methods for retrospective correction of these artifacts. A recent review of these methods has been written byTax et al. (2022). Machine learning methods have also been proposed to address these artifacts.

Much of the distortion correction algorithms for dMRI, such as those targeting susceptibility-induced distortions, are based on image registration. Image registration for dMRI data is especially challenging because of several factors: (1) low SNR, especially at higher b-values; (2) different appearance of dMRI volumes of the same brain acquired with different gradient strengths and directions, which is again exacerbated for higher b values; (3) difficulty of finding a reference image for registration since all dMRI volumes are affected by distortions; (4) complex interaction of various artifacts such as signal loss that can further challenge the registration process. Standard methods for distortion correction, such as topup (Andersson et al., 2003), use iterative optimization to compute the displacement field. Deep learning-based registration methods have the potential to address these challenges because they can be trained in an unsupervised manner on distorted data.

Unsupervised (Alkilani et al., 2023;Duong et al., 2020;Zahneisen et al., 2020), semisupervised (Legouhy et al., 2022), and supervised (Hu et al., 2020) deep learning-based registration methods have been applied on reversed phase-encoding dMRI data to estimate a displacement field for distortion correction. One study (Legouhy et al., 2022) combined unsupervised training with supervised training using the displacement fields computed by topup as the target. Experiments showed that this semisupervised method led to better results than purely supervised and purely unsupervised techniques. Most prior studies have shown that deep learning techniques match the accuracy of the conventional methods such as topup while being orders of magnitude faster (Alkilani et al., 2023;Duong et al., 2020;Legouhy et al., 2022;K. G. Schilling et al., 2020;Zahneisen et al., 2020). One study used distortion-free volumes obtained with point-spread function-encoded EPI as registration target and achieved more accurate distortion correction than topup (Hu et al., 2020). The input to the network, which was a 2D Fully Convolutional Network (FCN), consisted of distorted dMRI volumes as well as an anatomical T2-weighted image, and the network output was undistorted dMRI volumes. Machine learning methods also have good generalizability. Studies have shown that deep learning models trained on normal brains work well on abnormal brains (Hu et al., 2020;Zahneisen et al., 2020) and on different (left–right) phase-encoding directions (Zahneisen et al., 2020).

Most deep learning-based methods have used the dMRI volumes as input for computing the displacement fields for distortion correction.Qiao and Shi (2021)andQiao et al. (2019), however, propose to use fiber orientation distribution (FOD) as input. They argue that the information in the individual dMRI data volumes is not sufficient to resolve detailed white matter structures that are essential for distortion correction in areas such as the brainstem (Qiao et al., 2019). Instead, they compute FOD maps from dMRI volumes acquired with opposite phase encodings and train an FCN to compute the displacement fields. This method achieved better results than topup and two alternative deep learning methods in brainstem and white matter. Another interesting study is that ofK. G. Schilling, Blaber, et al. (2019)andK. G. Schilling et al. (2020), which enables susceptibility-induced distortion correction when scans with reversed phase encodings are not available. They have developed deep learning models to generate an undistorted b0 (nondiffusion-weighted) volume given (i) a distorted b0 volume and (ii) an anatomical (T1-weighted) image. The undistorted and distorted images can then be used to compute the displacement field with topup. Their methods are based on FCNs and generative adversarial networks (GANs;I. Goodfellow et al., 2020).

Deep learning methods have been developed to address other types of artifacts in dMRI.Ayub et al. (2020)have proposed a method based on variational autoencoders for in-painting cropped dMRI volumes. The model, trained on artificially cropped volumes and tested on real-world cropped data, was able to correctly inpaint the cropped data. Deep learning methods have been successfully applied to remove Gibbs artifacts in dMRI data. One study trained a CNN on T2-weighted images to estimate Gibbs ringing artifacts (Q. Zhang et al., 2019). The trained model removed Gibbs artifacts in dMRI volumes as well. Another study trained a CNN on natural images with simulated artifacts and showed that the trained model effectively suppressed Gibbs artifacts in dMRI data and in derived parameter maps such as fractional anisotropy (FA) images (Muckley et al., 2021).

#### 4.1.3 Automatic quality control

Quality control in dMRI data processing pipelines can be challenging because of the diversity of artifacts and large data sizes. A number of deep learning methods have been proposed for automatic quality control in large dMRI studies.Ahmad et al. (2023)trained a CNN to classify dMRI volumes as normal or containing artifacts. The artifacts considered in their study included motion-induced signal dropout, interslice instability, ghosting, chemical shift, and susceptibility-induced distortions. Their method had an accuracy of 92% on a pool of seven datasets with different acquisition protocols. Another study used a mix of real and simulated data to train a quality control method, based on CNNs, for detecting intravolume motion and signal dropout (Graham et al., 2018). To detect motion-corrupted volumes in neonatal dMRI volumes,Kelly et al. (2017)trained a random forest on the outputs of an ensemble of CNNs and achieved classification accuracies of well above 90%.Samani et al. (2020)also trained a CNN to detect a range of artifacts such as motion artifacts, ghosting, susceptibility-induced artifacts, and chemical shift. They reported a detection accuracy of 98% on multiscanner dMRI data. BothGraham et al. (2018)andSamani et al. (2020)used transfer learning to train their networks.

### 4.2 Quality enhancement, super-resolution, and data interpolation

Increasing the spatial resolution or q-space sampling density during image acquisition would require increasing the scan time. As an alternative, recent studies have proposed to use computational methods. Much of the proposed methodology have been inspired by deep learning-based super-resolution techniques. However, often these standard deep learning techniques have to be significantly modified to handle the dMRI data. A summary of these studies is provided below.

#### 4.2.1 Interpolation in q-space (i.e., angular space)

Several studies have used neural networks to enhance the angular resolution of q-space data (G. Chen et al., 2023;Koppers & Merhof, 2021;Lyon et al., 2023;Yin et al., 2019).Yin et al. (2019)used a method based on sparse representation of the dMRI signal and a neural network built on 1D convolutions to map low angular resolution data to high angular resolution data. This method improved the FOD reconstruction accuracy for complex fiber configurations. In another study,Ren et al. (2021)have developed a method for predicting dMRI data for arbitrary gradient directions. The input to this model includes only T1-weighted, T2-weighted, and b0 (nondiffusion-weighted) images. Methodologically, the model is a GAN, where both the generator and the discriminator are conditioned on the gradient direction and strength. Experiments are performed with training and test data from the Human Connectome Project (HCP). The results show that the synthesized data can be used to estimate DKI, Neurite Orientation Dispersion and Density Imaging model (NODDI;H. Zhang et al., 2012), and FOD.

Lyon et al. (2023)proposed a parametric continuous convolutional network for angular super-resolution of dMRI data. Their method improved the results of fixel-based analysis and estimation of NODDI. Another study has proposed a flexible model, based on a recurrent convolutional neural network (RCNN), that can predict the unobserved data for arbitrary target b-vectors (Lyon et al., 2022). To achieve this flexibility, the model follows an encoder–decoder design where the encoder uses the gradient table information of the measured data as additional input. The decoder, then, takes the latent representation generated by the encoder and the gradient table of the target (unobserved) data to predict the unobserved data. This model accurately upsampled the dMRI data by a factor of 8, and it was more accurate than predictions based on spherical harmonic interpolation and a 1D variant of the model architecture. At higher subsampling rates, spatial information study improved the accuracy of angular interpolation (Lyon et al., 2022). A more recent study proposed a geometric deep learning method to predict unobserved q-space data for arbitrary target acquisition schemes such as single/multishell and grid-based schemes (Ewert et al., 2024). Experiments showed that this method was superior to model-based (i.e., nonmachine learning) techniques and it improved the estimation accuracy for tissue microstructure and FOD.

#### 4.2.2 Interpolation in voxel space

Different from the methods above, a number of studies have attempted to increase the spatial resolution of the dMRI data in voxel space (Elsaid & Wu, 2019;Tian et al., 2021). It is also possible to jointly improve the spatial resolution and q-space resolution, as proposed in the super-resolved q-space deep learning (SR-q-DL) framework (Qin, Liu, et al., 2021). In this framework, patches from the source low-resolution and the target high-resolution dMRI volumes are represented in dictionaries and a neural network is trained to map the representation of the source signal to that of the target signal. This method significantly improved the accuracy of estimating the NODDI parameters from low-quality dMRI data. In a more recent work,Spears and Fletcher (2023)have argued that prior voxel space super-resolution methods such asQin, Li, et al. (2021)are not ideal for tractography, where a continuous FOD field is desired. Instead, they have proposed a method based on continuous and differentiable signal representations with neural networks. They show that their new method achieves state-of-the-art FOD estimation and tractography on HCP data.

#### 4.2.3 Enhancement of derivative maps

A number of studies have proposed to enhance the quality/resolution of the derived parameter maps.Zeng et al. (2022)developed a method to enhance the resolution of FOD maps. Their method used the FOD estimated from low angular resolution single-shell data to predict the FOD estimated from multishell high angular resolution data. Experiments on clinical quality data showed that this method significantly improved the tractography and structural connectivity assessment. A similar study has been reported byLucena et al. (2021), where the authors trained CNNs to map the FODs computed from single-shell data to those computed from multishell data. The method worked well and showed good generalizability to different scanners and data acquisition protocols.Ye, Qin, et al. (2019)proposed to combine a learning-based parameter mapping method similar toYe (2017a)with a super-resolution method similar toTanno et al. (2017)in a joint framework to estimate high-resolution NODDI maps from dMRI measurements with low resolution in both voxel space and q-space.

#### 4.2.4 Image quality transfer

In a series of studies,Alexander et al. (2017;Blumberg et al., 2018) have developed and promoted the notion of image quality transfer (IQT), which uses machine learning methods such as random forests and neural networks to improve the quality of dMRI parameter maps. The goal of IQT is to use high-quality data (such as the HCP data) to improve the quality of regular dMRI data that are typically obtained in clinical applications. The method works by learning a regression function to map the low-quality dMRI patches to high-quality patches. This idea has been successfully applied to increase the spatial resolution of diffusion tensor images and to estimate NODDI parameters from low-quality single-shell measurements. Furthermore, it has been shown that on low-quality data, IQT enables tractography-based reconstruction of thin fiber tracts that can typically only be reconstructed using specialized data.

#### 4.2.5 Estimation/imputation of unobserved or missing data

Estimating unobserved q-space shells from observed ones has been reported by several studies (G. Chen et al., 2023;Koppers, Haarburger, & Merhof, 2017;Murray et al., 2023).Murray et al. (2023)used neural networks to predict multishell data from single-shell data and used the predicted data to estimate the NODDI model. They achieved satisfactory results on healthy brains as well as brains of MS patients. In a similar work,G. Chen et al. (2023)successfully trained a multilayer perceptron (MLP) to predict six-shell data from two-shell data. A closely related application is imputation of missing data, which is of particular importance because some dMRI measurements often have to be discarded due to strong noise, artifacts, or excessive motion, such as in young or noncooperative subjects. To address this problem,Hong, Chen, et al. (2019)developed a method based on graph CNNs. They represented the relation between the measurements in the voxel space and q-space using a graph, and trained a residual graph CNN to learn this relation. They trained the model using adversarial techniques and successfully applied it on data from healthy infants between 0 and 12 months old. This method was able to reconstruct accurate maps of generalized FA (GFA) and FOD from five-fold-reduced slice acquisitions.

### 4.3 Data harmonization

Data harmonization is crucial for reliable analysis and comparison of interscanner and intersite dMRI data. It has become an increasingly more relevant problem as larger and geographically/demographically more diverse datasets are used in multicenter neuroimaging studies. Statistical methods such as ComBat (Fortin et al., 2017) and RISH (Mirzaalian et al., 2016) have achieved considerable success in addressing this problem. However, they have important limitations. For example, they depend on data from matched subjects in the reference and target datasets, and their performance deteriorates when the differences between reference and target datasets are nonlinear (Cetin-Karayumak et al., 2020).

Recently, various machine learning-based methods have been proposed, but they still have not achieved the popularity of methods such as ComBat and RISH. A recent study compared a range of interpolation methods, statistical regression techniques, and deep learning methods for cross-scanner and cross-protocol multishell dMRI data harmonization (Ning et al., 2020). A regression method based onKarayumak et al. (2019)performed better than all deep learning methods. However, some of the deep learning methods were among the best performing techniques.Ning et al. (2020)hypothesized that the performance of the deep learning methods may significantly improve with larger training datasets than the 10 training subjects used in that work.Tax et al. (2019)evaluated five different learning-based harmonization methods (including four neural networks and a dictionary-based technique) on data from different scanners and with different maximum gradient strengths. Their results showed that overall these learning-based methods were successful in harmonizing cross-scanner and cross-protocol data, although no comparison with the state-of-the-art nonlearning methods was performed. Their analysis also showed that the learning-based methods were more effective on isotropic measures such as MD than on anisotropic measures such as FA. They attribute this behavior to the possibility that, because the spatial variations in isotropic measures are less abrupt, imperfect spatial alignments may be less harmful when applying machine learning methods on imperfectly registered pairs of dMRI volumes.

Table 1lists some of the existing machine learning methods for dMRI data harmonization. In general, these methods offer higher flexibility in modeling the sources of heterogeneity and in handling data from unpaired subjects. For example, one study developed a method based on representing the data in a disentangled latent space that allowed for separating the effects of anatomy and acquisition (Hansen et al., 2022). Similarly, variational autoencoders have been proposed to harmonize dMRI data by learning a latent representation that is invariant to site/protocol-specific factors (Moyer et al., 2020). Focusing on the specific task of FOD estimation, another study developed a method, named null space deep network, that seamlessly integrated a small dMRI dataset with histology ground truth and a large scan–rescan dMRI dataset without histology ground truth (Nath, Parvathaneni, et al., 2019). The method showed superior FOD estimation accuracy on data from scanners that had not been included in the training set. A similar method was applied to harmonize structural connectivity metrics on a two-center dataset (Newlin et al., 2023). The authors found that optimal harmonization was achieved when the deep learning method was applied to harmonize the dMRI data at a voxel level followed by applying ComBat (Fortin et al., 2017) to harmonize the structural connectivity metrics. Another study from the same research team, again focusing on FOD prediction, proposed an extra loss term to encourage consistency of FOD estimations computed from scan–rescan data of the same subject (Yao, Rheault, Cai, Asad, et al., 2023). This new loss function improved the generalizability of FOD estimation as well as downstream tasks such as structural connectivity assessment.Blumberg et al. (2019)have proposed a multitask learning strategy, where a neural network is trained to combine the information learned by an ensemble of different neural networks. The individual neural networks in the ensemble may have been trained on separate datasets, possibly for completely different tasks. The added neural network model uses the high-level features learned by the ensemble to perform data harmonization. The authors argue that this approach can be especially effective in scenarios where one or some of the datasets are very small.

**Table 1.** A listing of some of the machine learning-based methods for data harmonization in dMRI.

Method	Summary of methodology and results
Semi-supervised contrastive learning using CNNs ( Hansen et al., 2022 )	This method uses paired data to learn subject-specific and acquisition-specific representations. A decoder is trained to map the subject-specific representations to the target contrast. The method is shown to be superior to a method based on CycleGAN ( Zhu et al., 2017 ) as well as interpolation based on SHORE ( Ozarslan et al., 2009 ). It can handle heterogeneous data from multiple sites, different acquisitions protocols, and demographics.
Null space learning with an MLP architecture ( Nath, Parvathaneni, et al., 2019 )	The model is trained using paired dMRI scans of human subjects as well as dMRI-histology data from squirrel monkeys. The loss function is designed to encourage accurate FOD estimation and consistent scan–rescan GFA. Experiments show that this method has high accuracy in FOD estimation and high reproducibility on test data from unseen scanners. Experiments also show that this approach results in higher FOD estimation accuracy across 1.5T and 3T scanners.
Variational autoencoder ( Moyer et al., 2020 )	The method is based on learning a latent representation that is invariant to scanner, scanning protocol, or similar confounders. The latent representation can then be used to reconstruct the image content that is stripped of those factors. The method can be trained without paired scans. Training involves an adversarial loss that attempts to predict the source (scanner, etc.) of the acquisition. Results show that, compared with a well-established conventional technique ( Mirzaalian et al., 2018 ), this new method achieves superior results in terms of several parameters including FA, MD, and fiber orientation.
Residual CNN ( Koppers et al., 2019 )	This method is based on predicting the spherical harmonics representation of the dMRI signal in the target domain from that of the source domain. It works on 3D patches of size 3. A final nonlearned projection in the spherical harmonics space is needed to make sure fiber orientations are not changed due to the intensity harmonization. This method requires that the same subjects be scanned in the source and target scanner/acquisition protocol. Evaluations have shown that this method can achieve effective harmonization of the dMRI signal, FA, and MD.
Hierarchical CNNs ( Tong et al., 2020 )	The focus of this work is on harmonization of DKI measures. The method requires a set of subjects to be scanned with both target and source scanners/acquisition protocols. It computes the DKI metrics using the data in the target domain. The network is trained to map the dMRI data in the source domain directly to the DKI measures in the target domain, after nonlinear intrasubject registration. This method reduced the interscanner variation in DKI measures by 51–66%.
CNN with a scan–rescan consistency loss ( Yao, Rheault, Cai, Nath, et al., 2023 )	This method is proposed and evaluated specifically for FOD estimation, although it seems to be directly applicable for estimating any other parameter. A consistency loss penalizes the model for divergent FOD predictions from different scans of the same subject. It achieves better FOD estimation accuracy and interscanner consistency than standard techniques on external test data.
Ensemble of different neural networks ( Blumberg et al., 2019 )	The authors present their method as a multitask learning approach. Essentially, a set of neural networks, which can have different architectures and trained for different prediction tasks, are combined via training a set of additional neural networks that utilize the features learned by these networks to predict the parameter of interest. Compared with state-of-the-art deep learning techniques, this method achieved better dMRI signal prediction.
Adaptive dictionary learning ( St-Jean et al., 2020 )	A dictionary is learned on the set of reference datasets/scanner(s). It is assumed that representing a test scanner’s data in this dictionary automatically harmonizes the data toward the reference dictionary by suppressing the features that are specific to the test data. The method does not require paired subjects in the source and reference datasets. Successful harmonization results are reported in terms of FA, MD, and rotationally invariant spherical harmonics representations.

### 4.4 Microstructure mapping

The brain white matter consists of a network of neuronal fibers that are supported by other cellular components (Edgar & Griffiths, 2009;Novikov et al., 2019). Microstructural properties of the white matter tissue (e.g., myelination) influence its physical properties such as viscosity and permeability (Kuroiwa et al., 2006;Mellergård et al., 1989). These physical properties, in turn, alter the diffusion of water molecules in the tissue. Although the size of the microstructural elements is in the micrometer range, dMRI measurements are sensitive to the displacement of water molecules at the micrometer scale (Assaf & Cohen, 2014;Le Bihan, 2014). Therefore, the dMRI signal is an indicator of the changes or variations in brain tissue microstructure that can arise due to normal development, aging, or injuries and diseases (Bodini & Ciccarelli, 2009;Bozzali & Cherubini, 2007;Calixto, Soldatelli, et al., 2024;Lakhani et al., 2020;Salat, 2014).

There have been many efforts and much progress in developing biophysical models that relate the measured dMRI signal to the microstructure of brain tissue (Novikov et al., 2018,^2019^). Advanced models rely on specialized measurement protocols and complex numerical optimization methods. The underlying estimation problem is typically nonlinear, sensitive to measurement noise and initialization, and may be unstable and computationally intensive (Harms et al., 2017;Jelescu et al., 2016). Accuracy and precision of model fitting are hard to assess and may depend on the optimization algorithm used in fitting (Harms et al., 2017). To avoid local minima, some studies have resorted to computationally intensive approaches such as grid search, multistart methods, cascade optimization, and stochastic optimization techniques (Alexander, 2008;Alexander et al., 2010;Harms et al., 2017;Jelescu et al., 2016). There is a great interest in developing methods, such as those based on dictionary matching (Daducci et al., 2015;Rensonnet et al., 2019), to reduce the computational time without compromising the estimation accuracy. A good example of such methods is AMICO (Daducci et al., 2015), which reformulates the microstructure estimation equations as convex optimization problems that can be solved much faster.

#### 4.4.1 Potential advantages of machine learning methods

In recent years, machine learning has increasingly been applied to these estimation tasks. Overall, five main justifications have been cited for preferring a machine learning-based approach for this application.

1. Unlike the conventional methods that presume a known fixed relationship between the dMRI measurements and the target parameter, machine learning methods can learn this relationship from data (Fick et al., 2017;Golkov et al., 2016). For certain microstructural parameters, such as residence time of water inside axons, it is believed that existing mathematical models that express the relationship with the dMRI signal are either too simplistic or intractable, and that the existing numerical forward models are computationally too expensive to be used in estimating the parameters from data (Hill et al., 2021;Nedjati-Gilani et al., 2017).Fick et al. (2017)show that, for axonal diameter estimation, existing signal models fail outside a narrow range of diameters, while a machine learning method can achieve accurate estimation for the whole range of diameters in the data.
2. Conventional methods often involve several steps with no feedback from the later steps to the earlier steps (Golkov et al., 2016;Harms et al., 2017). As a result, these methods are hard to design and optimize. Machine learning methods such as deep neural networks, however, may be optimized end-to-end as a single processing step.
3. Machine learning methods can be much faster than numerical optimization routines. Again, such is the case for neural networks that run on GPUs (Barbieri et al., 2020;de Almeida Martins, Nilsson, Lampinen, Palombo, While, et al., 2021;Kaandorp et al., 2021).
4. Standard methods perform the model fitting in a voxel-wise manner, which fails to exploit the spatial correlations to improve the estimation accuracy. Machine learning models can effectively learn complex spatial correlations directly from data and leverage this knowledge to improve the estimation accuracy (Gong et al., 2023).
5. With machine learning methods, it is typically much easier to incorporate prior knowledge or additional information such as constraints on the parameters to be estimated or other MRI contrasts.

Tables 2,3, and4list some of the recent studies that have employed machine learning to estimate, respectively, DTI, DKI, and NODDI parameters. A summary of the methods developed in these studies and their experimental results is presented below.

**Table 2.** Machine learning methods for estimating the diffusion tensor or its derived parameters such as FA and MD.

Method	Summary of methodology and results
SuperDTI ( H. Li et al., 2021 )	This work reports accurate estimation of FA, MD, and the main diffusion tensor eigenvector from six measurements. The model trained on healthy HCP brains works well on pathological brains. Evaluations included qualitative and quantitative tractography assessment.
DeepDTI ( Tian et al., 2020 )	This method trains a CNN to compute *dMRI data residuals* . Specifically, it computes the residuals between undersampled ( n=6 ) data and high-quality targets computed from densely sampled data. The diffusion tensor is then computed with a standard method. Compared with conventional estimation techniques, this method reduced the number of measurements by a factor of 3.3–4.6. It improved DTI estimation, DTI-based tractography, and tract-specific analysis on 20 prominent tracts. Only HCP data are used for validation.
Aliotta et al. (2019)	This study reported higher FA and MD reconstruction accuracy and precision than standard estimation methods from as few as three diffusion-weighted measurements. With a model trained on data from 10 healthy subjects, FA-based delineation of brain tumors was more accurate than with a standard method.
DIFFnet ( Park et al., 2021 )	The main contribution of this study is a method to handle the measurements acquired with different schemes. The method simply projects and bins the the q-space data in standard orthogonal planes. Compared with standard methods, they report faster computation and lower error.
Fetal DTI estimation ( Karimi, Jaimes, et al., 2021 )	This study has reported reconstruction of fetal DTI with unprecedented accuracy. The method was trained using synthetic data generated with a novel pipeline that used both fetal in utero data and scans of premature neonates. Evaluations included quantitative comparisons with conventional methods as well as detailed assessment by human experts.
Patch-CNN ( Goodwin-Allcock et al., 2023 )	The proposed method, a patch-wise CNN, reduces the required number of measurements by a factor of 2 compared with standard estimation methods. The estimated DTI maps were used to trace major white matter tracts with high accuracy.
Transformer-based DTI estimation ( Karimi & Gholipour, 2022b )	This study used transformer networks to learn the spatial correlations in dMRI signal and in diffusion tensor. The method reconstructed the diffusion tensor with superior accuracy while reducing the required number of measurements by factors of 5–15. Evaluations include tractography and structural connectivity.
Cardiac DTI estimation (FG-Net) ( S. Liu et al., 2023 )	This study has achieved accurate cardiac DTI estimation from six dMRI measurements. It includes a basic FCN that estimates the dMRI data for additional directions than the six measured. The method predicts DTI metrics more accurately than conventional methods on ex vivo data.
Cardiac DTI ( Weine et al., 2022 )	The model (a residual CNN) trained with purely synthetic data performs well on synthetic as well as in vivo test data for cardiac DTI estimation. It is less prone to predicting implausible values and enables more accurate detection of tissue lesion.
SwinMR (cardiac DTI) ( Huang et al., 2024 )	Deep learning methods can reconstruct cardiac DTI with k-space undersampling rates of up to 4 without any significant quality reduction compared with reference. A transformer network is shown to achieve superior results than a CNN.

**Table 3.** A summary of recent machine learning techniques for estimating the parameters of the NODDI model.

Method	Summary of methodology and results
DLpN ( Faiyaz et al., 2022 )	A dictionary learning method is first used to estimate the isotropic volume fraction. Subsequently, a neural network computes the remaining NODDI parameters. This method estimates the NODDI parameters from a single-shell (b = 2000) acquisition as accurately as conventional methods with multishell data. The method is also validated on clinical data.
Deep sparse representation methods ( Ye, Li, & Chen, 2019 ) ( Ye et al., 2020 )	These related studies compute sparse representation of the signal in dictionaries. Sparse representation coefficients are then used by neural networks to predict the microstructure indices. Using only HCP data, more than twice reduction in estimation error compared with optimization methods is reported.
MEDN ( Ye, 2017c )	This method is based on representing the dMRI signal in a dictionary-matching framework inspired by AMICO ( Daducci et al., 2015 ) and related to the above two methods ( Ye, Li, & Chen, 2019 ; Ye et al., 2020 ). An MLP-type network is used to compute the NODDI parameters from the signal representations. The method achieves more than twice lower estimation error and faster computation compared with standard techniques.
Gibbons et al. (2019)	This study simultaneously estimated NODDI and generalized FA. The trained model worked well on data from healthy individuals and stroke patients. The method is a basic 2D FCN.
G. Chen et al. (2023)	This study used MLP models to predict unmeasured dMRI signal and microstructure indices including NODDI parameters. New loss functions are introduced to encourage accurate prediction of the dMRI signal and microstructure indices. Using only HCP-style data, the effectiveness of the new loss functions is demonstrated. No comparisons with standard estimation methods are reported.
METSC ( Zheng et al., 2023 )	This study makes use of sparsity-based representation of the dMRI signal, computed using an unfolded iterative hard thresholding method, and a transformer network. It achieves an 11-fold reduction in the required number of measurements compared with conventional methods and reduces the estimation error compared with other learning-based methods.
q-space deep learning ( Golkov et al., 2016 )	Using a voxel-wise estimation with an MLP, this study reported a 12-fold reduction in the required number of measurements to achieve the same level of accuracy as standard methods. As few as eight measurements were sufficient for accurate estimation. Four different datasets including one from MS patients are used.
Machine learning-informed estimation ( Gong et al., 2023 )	The focus of this study is on very low SNR scenarios such as spinal cord imaging. An MLP is used to estimate good initial values for the NODDI parameters. Refined estimates are subsequently computed using a maximum likelihood estimation technique.
SR-q-DL ( Qin, Liu, et al., 2021 )	This method follows a super-resolution framework. Specifically, the low-resolution dMRI signal volumes are used to compute high-resolution tissue microstructure maps. The method itself has two stages. The first stage learns a sparse representation of the dMRI signal with 1D convolutions. The second stage is a CNN that performs the computation. Evaluations have shown that this method outperforms conventional and machine learning methods. However, only HCP data are used to develop and validate the method.
DIFFnet ( Park et al., 2021 )	This is a CNN-based method that achieves low reconstruction error while reducing the computation time by three orders of magnitude.
Population-based Bayesian regularization ( Mozumder et al., 2019 )	This is a Bayesian estimation method, where a population-informed prior is computed from a cohort of healthy subjects and included in the estimation for test subjects. Results show that the use of the population-based prior helps alleviate the ill-posedness of the estimation problem and significantly improves the estimation accuracy and robustness. The model was developed using HCP data and validated on an independent dataset.

**Table 4.** A summary of recent machine learning methods for DKI estimation.

Method	Summary of methodology and results
Separable dictionaries and deep learning ( HashemizadehKolowri et al., 2022 )	A comparison of different deep learning methods showed that a technique that utilized separable dictionaries ( Ye et al., 2020 ) achieved the best results for jointly estimating DTI, DKI, and NODDI parameters. Only HCP data were used. Moreover, the deep learning methods show some systematic biases, such as underestimation of radial kurtosis.
q-space deep learning ( Golkov et al., 2016 )	This study has reported accurate estimation of DKI parameters from 12 measurements. The model is a voxel-wise MLP, which is tested in multiple external datasets.
Z. Li et al. (2019)	The method estimates DTI and DKI metrics jointly. Results show that DKI parameters can be estimated with eight measurements.
Masutani (2019)	This study trained an MLP using synthetic data. Only four diffusion-weighted measurements were used for DKI estimation. Tests on synthetic and real data showed that high estimation accuracy was achieved. This study also demonstrated the importance of noise level matching between training and test datasets.
Hierarchical CNNs ( Gong et al., 2021 )	The deep learning model is a hierarchical CNN that is combined with a motion detection and rejection technique. Experiments, including data from children with attention deficit hyperactivity disorder, show that the new method is robust to head motion and can estimate the DKI parameters from eight measurements. It works well in the presence of severe motion, when up to 90% of the data are motion corrupted.
MLP + least squares ( Kerkelä, Seunarine, Henriques, et al., 2022 )	This study uses an MLP to estimate initial DKI parameter values, which are then used within a regularized least-squares procedure to compute the final values. This approach avoids implausible predictions and improves model robustness compared with existing linear and nonlinear optimization methods. The data include scans of 10 healthy volunteers.

#### 4.4.2 Method design

Methodologically, these studies have been dominated by neural networks, although other models such as random forests (Fick et al., 2017;Hill et al., 2021;Nedjati-Gilani et al., 2017) and Bayesian estimation methods (Mozumder et al., 2019;Reisert et al., 2017) have also been used. The first study to propose deep neural networks for this purpose was q-space deep learning (Golkov et al., 2016). It reported accurate estimation of DKI parameters with 12 measurements and NODDI parameters with 8 measurements, resulting in a 12-fold reduction in scan time. Many studies have followedGolkov et al. (2016)to develop neural network models for tissue microstructure mapping. This should not be surprising given that deep neural networks have emerged as the best-performing regression models in many applications (Lathuilière et al., 2019). Most nonneural network methods either predate the recent surge of deep learning or do not include a rigorous comparison with neural networks.

Incorporation of population-estimated priors has been advocated by several studies (Karimi & Gholipour, 2022a;Mozumder et al., 2019). For estimation of NODDIDA (NODDI with Diffusivity Assessment), it was shown that estimating a multivariate Gaussian prior from 35 subjects significantly improved the prediction accuracy and robustness (Mozumder et al., 2019). The authors concluded that incorporation of the prior reduced the ill-posedness of the estimation problem and made it possible to estimate this complex model from clinically feasible measurements. Some studies have suggested utilizing other inputs in addition to the dMRI measurements. For instance, one study used T1-weighted and T2-weighted images (registered to the dMRI data) to improve diffusion tensor estimation (Tian et al., 2020).

#### 4.4.3 Training procedures and data

Most studies have adopted an end-to-end training approach, where the input dMRI measurements are mapped to the target parameter of interest using a single (albeit deep) neural network. However, there are notable exceptions that are instructive. For DKI,Kerkelä, Seunarine, Henriques, et al. (2022)used the neural network predictions as input to a regularized nonlinear least-squares method to compute the final values. This approach improved the estimation robustness of the standard method and reduced the probability of predicting implausible values. Other studies have also proposed to combine neural network estimators with conventional methods. For example, for computing the NODDI parameters under very low signal-to-noise ratio (SNR),Gong et al. (2023)used neural networks to obtain good initial estimates, which were then used to obtain more accurate estimates using maximum likelihood estimation (MLE). The justification for this approach is that when SNR is low, MLE is prone to erroneous predictions because of its vulnerability to local minima. The predictions of the neural networks, in contrast, although very fast, can suffer from small but significant biases. Hence, one can use the neural network to compute a good initial estimate, which can then be refined using an unbiased MLE estimator. A similar approach was used inFaiyaz et al. (2022), where a shallow neural net was used to estimate the isotropic volume fraction in the NODDI model, which was used in a MLE formulation to compute the complete NODDI model from single-shell data.

For diffusion tensor estimation from six measurements,S. Liu et al. (2023)argue that directly mapping the measurements to the tensor is not optimal. Given the measurements along six gradient directions, they employ a neural network to estimate the signal along additional directions. These measurements are subsequently used by a second network to estimate the diffusion tensor.Ye (2017c);Ye, Li, and Chen (2019)have proposed methods based on sparse representations and deep neural networks. In one implementation, their method is a two-stage pipeline: the first stage uses an LSTM model to compute a sparse decomposition of the signal in a dictionary. An MLP computes the desired microstructure indices based on the sparse representation coefficients. This study has been in part inspired by the widely used AMICO method (Daducci et al., 2015), which decouples the estimation of NODDI and ActiveX (Alexander et al., 2010) models into linear problems and solves them using sparsity-based optimization.Ye et al.’s (2020)experiments show that this method can estimate NODDI parameters with higher accuracy than standard estimation techniques. This method was further improved with the help of a separable spatial–angular dictionary for signal representation.

The most common training approach has been supervised training, where the loss function to be minimized is computed based on the difference between the predicted and ground truth parameter values. However, there have been important and instructive exceptions.Kaandorp et al. (2021,^2023^) propose to train a neural network model in an unsupervised manner by using the predicted tissue parameters to predict the corresponding dMRI signal and optimizing the network weights to minimize the difference between the predicted and measured signal. They found that predictions of unsupervised methods had higher variability, whereas predictions of supervised methods had a strong bias toward the mean of training data and were deceptively smooth (Kaandorp et al., 2023).Epstein et al. (2022)have analyzed the bias-variance trade-off in supervised and self-supervised microstructure estimation methods. They argue that the reason for the lower bias of self-supervised methods is because they are based on the same optimization objective as MLE, while the high bias in the supervised method is because they deviate from this objective and are based on a poor choice of training target. They show that using an MLE-computed parameter value as the estimation target can reduce the estimation bias of supervised methods.

Most commonly, target microstructure values for the training data are computed by applying a standard estimation method on dMRI measurements. This is often justified by using densely acquired high-SNR measurements to improve the accuracy of this computation. Then, the machine learning model is trained with downsampled measurements to match the estimation accuracy of the standard method. However, this approach inevitably inherits some of the limitations of the standard method that is used to compute the prediction target. Very little attention has been paid to this issue (Zucchelli et al., 2021a). One study proposed to inspect the results computed by the standard fitting method and remove the voxels that contain implausible values (Kerkelä, Seunarine, Henriques, et al., 2022).

In some applications, obtaining reliable in vivo training data may be hard or impossible. Two examples are cardiac DTI (Weine et al., 2022) and fetal imaging (Karimi, Jaimes, et al., 2021). For cardiac DTI,Weine et al. (2022)developed a parameterized pipeline to synthesize training data with realistic spatial correlations and cardiac motion. For fetal brain DTI, another study proposed a pipeline that synergistically combined data from preterm neonates and fetuses to synthesize realistic fetal dMRI data (Karimi, Jaimes, et al., 2021). Both studies reported good results on independent real test data. Using synthetic data has the added advantage that it can incorporate a much wider range of parameters and factors such as noise and motion than can be available in any in vivo dataset.

#### 4.4.4 Evaluations and main results

Overall, the results of the published studies suggest that machine learning methods may be capable of achieving higher estimation accuracy than conventional methods (Tables 2,3, and4). They may also be able to reduce the required number of measurements. Some studies have found that estimation accuracy of deep learning methods may be less affected by suboptimal acquisition protocols than numerical optimization methods (de Almeida Martins, Nilsson, Lampinen, Palombo, While, et al., 2021). There are, however, studies that have reported contrary results that deserve careful consideration. For example, one study has reported that neural network and random forest models trained on simulated data may produce less accurate and more biased results compared with nonlinear least-squares estimation methods (Gyori et al., 2022). Another study has shown that deep learning methods can achieve good estimation accuracy when the optimization landscape is well behaved but that they have poor performance when the optimization landscape is degenerate such as for multicompartment models with even two compartments (de Almeida Martins, Nilsson, Lampinen, Palombo, Westin, & Szczepankiewicz, 2021;Mozumder et al., 2019).

It has also been reported that machine learning methods are less sensitive to measurement noise and other imperfections (Gong et al., 2019;Jung et al., 2021). As an example,Gong et al. (2019)trained a deep learning model to compute DTI and DKI parameters using data from healthy subjects with voluntary head motion as well as attention deficit hyperactivity disorder (ADHD) patients with nonvoluntary motion. They found that predictions of the deep learning model were less sensitive to motion and comparable with predictions with motion-free data.

It has been shown that models that leverage spatial correlations in a neighborhood (e.g., an image patch) around the voxel of interest can lead to more accurate estimation (Aliotta et al., 2021;Gibbons et al., 2019;HashemizadehKolowri et al., 2022;H. Li et al., 2021). Early studies used CNNs to learn the spatial correlations. More recent studies have relied on attention-based neural networks that are considered to be more effective in learning correlations (Karimi, Afacan, et al., 2020;Karimi, Jaimes, et al., 2021;Zheng et al., 2023).Zheng et al. (2023)argue for the superiority of transformer architectures for tissue microstructure mapping in dMRI. In order to enable effective implementation of these architectures with small datasets, they introduce additional computational modules. Specifically, they use a sparse representation stage to compute the signal embeddings in a dictionary using unfolded iterative hard thresholding.

Although most studies have evaluated the new methods only in terms of estimation accuracy measures such as root mean square of the error (RMSE), several studies have investigated the downstream use of the estimated tissue microstructure parameters. For example,Gibbons et al. (2019)showed that poststroke outcome prediction was the same for microstructure maps estimated with standard methods and those estimated with a neural network with 10-fold fewer measurements.Aliotta et al. (2019)observed that deep learning-estimated FA maps resulted in more accurate delineation of the brain tumor boundaries in glioblastoma multiforme patients than with conventional methods.Kaandorp et al. (2021)showed that intravoxel incoherent motion (IVIM) parameters computed by a deep learning method better predicted the chemoradiotherapy response of pancreatic cancer patients than a standard method. Another study assessed the microstructure maps estimated by several deep learning methods in terms of their effectiveness in studying the impact of migraine on the brain white matter (Aja-Fernández et al., 2023). Specifically, FA and mean diffusivity (MD) values computed by different methods were compared using Tract-Based Spatial Statistics (TBSS) (S. M. Smith et al., 2006). It was observed that, compared with a standard estimation technique, deep learning methods improved the true positive rate but also increased the false positive rate.

For DTI estimation, several studies have reported good reconstructions from the theoretical minimum of six measurements (Table 2). DeepDTI (Tian et al., 2020) uses six diffusion-weighted measurements and a nonweighted (b0) measurement, T1-weighted, and T2-weighted images as input and computes the residuals with respect to high-quality measurements. The estimated high-quality measurements are then used to estimate the diffusion tensor with a standard estimation technique. Detailed analysis, including local assessment of DTI-derived parameters, tract-based analysis, and tractography show that DeepDTI achieves accurate estimation while reducing the number of measurements by a factor of at least 3–5. DeepDTI is based on a 3D FCN and requires that the six diffusion-weighted measurements be acquired along the optimal directions proposed inSkare et al. (2000). SuperDTI, proposed byH. Li et al. (2021), also used an FCN for direct estimation of the diffusion tensor. It achieved accurate FA computation and tractography with six measurements and accurate MD computation with three measurements. Similar results were reported inAliotta et al. (2019,^2021^). Using transformer networks, another study made a similar claim and further evaluated the method using tractography and quantitative structural connectivity assessment (Karimi & Gholipour, 2022b). DiffNet (Park et al., 2021) estimated FA and MD with 3–20 diffusion-weighted measurements and showed that the estimation accuracy was still higher than standard methods. DiffNet-estimated FA was more accurate for segmenting brain tumor volume, while also reducing the scan time.

#### 4.4.5 Other dMRI-derived parameters

In addition to the DTI, DKI, and NODDI parameters, machine learning methods have also been applied to compute other parameters. Examples include myelin water fraction (Fick et al., 2017;Jung et al., 2021), water residence time in white matter (related to axonal permeability) (Hill et al., 2021;Nedjati-Gilani et al., 2017), axonal radius (Fick et al., 2017;Nedjati-Gilani et al., 2017), spherical mean technique (SMT) (Gyori et al., 2022;HashemizadehKolowri et al., 2022;Ye, Li, & Chen, 2019), ensemble average propagator (EAP) models (Ye, 2017b;Ye, Li, & Chen, 2019), and relaxation–diffusion models of white matter microstructure (de Almeida Martins, Nilsson, Lampinen, Palombo, While, et al., 2021;de Almeida Martins, Nilsson, Lampinen, Palombo, Westin, & Szczepankiewicz, 2021;Grussu et al., 2021).Pirk et al. (2020)developed a novel magnetic resonance fingerprinting sequence to simultaneously estimate T1-weighted, T2-weighted, and diffusion tensor parameters. Instead of the standard dictionary matching approach, they used a deep neural network to estimate the target parameters and achieved good results in healthy brains and brains of MS patients.

A number of studies have attempted to use deep neural networks to estimate the IVIM model (Barbieri et al., 2020;Bertleff et al., 2017;Epstein et al., 2022;Kaandorp et al., 2021,^2023^;Parker et al., 2023;L. Zhang et al., 2019;Zheng et al., 2023). One study showed that an MLP could achieve results that were comparable with or more accurate than least-squares estimation and Bayesian techniques (Barbieri et al., 2020). Another study found that a neural network could outperform the state-of-the-art estimation methods for computing the parameters of a combined IVIM-kurtosis imaging model (Bertleff et al., 2017). It also showed that the neural network method made fewer predictions that were outside the range of plausible parameter values. One study showed that a neural network, trained using an unsupervised approach, produced more consistent and more accurate estimations than standard methods and it better predicted the chemoradiotherapy response of pancreatic cancer patients (Kaandorp et al., 2021).

### 4.5 Fiber orientation density estimation

Estimation of fiber orientation distribution (FOD), especially in regions with complex fiber configurations, requires high angular resolution diffusion imaging (HARDI) measurements. However, even when such measurements are available, this is a challenging estimation task. Recent studies have highlighted the inherent limitations of FOD estimation based on dMRI data (K. Schilling et al., 2016;K. G. Schilling et al., 2018). Nonetheless, the advantages of estimating crossing fiber configurations for applications such as tractography and connectivity analysis have been shown time and time again (Bucci et al., 2013;Prčkovska et al., 2016). As a result, there have been ongoing efforts to improve the accuracy of FOD estimation. The simplest approach to characterizing crossing fibers may be the multitensor model (Peled et al., 2006;Tuch et al., 2002). However, these methods require determination of the number of tensors in each voxel, which is a difficult model selection problem (Scherrer et al., 2013;Schultz et al., 2010). At present, spherical deconvolution methods such as constrained spherical deconvolution (CSD) are the most widely used techniques for assessing crossing tracts (Jeurissen et al., 2014;Tournier et al., 2007). These methods consider the dMRI signal in q-space to be the result of the convolution between a spherical point-spread function representing the fiber response function and the FOD. Naturally, they estimate the FOD via deconvolution of the dMRI signal with this response function.

Many machine learning methods have been proposed for FOD estimation.Table 5provides a brief listing of some of these methods. We summarize our main observations and conclusions from our study of these studies below.

**Table 5.** A summary of some of the machine learning methods for FOD estimation.

Method	Summary of methodology and results
Autoencoder-based FOD regularization ( Patel et al., 2018 )	This study uses autoencoders to learn FOD priors from high-quality dMRI data. The prior is used to constrain CSD for FOD estimation on test data. Compared with standard estimation techniques, the new method reduces the required number of measurements by a factor of 2 and can work with a lower diffusion strength (b-value). The method is tested on only one subject.
Equivariant networks ( Elaldi et al., 2021 ), ( Elaldi et al., 2023 )	These studies are based on rotation- and translation-equivariant convolutional networks. Training is performed in an unsupervised manner by convolving the computed FOD with the tissue response function to predict the dMRI signal and using the error in the predicted signal as optimization loss. The new methods result in lower FOD estimation error and more accurate tractography on in vivo human data and phantom data.
MLP trained with histology ground truth ( Nath, Schilling, et al., 2019 )	These studies have developed and validated FOD estimation models using training data with histology-derived FOD ground truth. The models are MLPs with residual connections. Results show that the deep learning methods lead to higher estimation accuracy (quantified in terms of angular correlation coefficient) compared with standard methods such as CSD. On in vivo human data, the new method shows higher reproducibility.
CNN method for fetal and neonatal brains ( Kebiri et al., 2024 )	A CNN has been used to predict FOD from six diffusion-weighted measurements for neonatal brains. Estimation accuracy is on par with the state of the art on neonatal scans. The method also performs well qualitatively on out-of-distribution clinical datasets of fetuses and newborns.
CNN classifiers ( Koppers & Merhof, 2016 )	The method, which is based on CNN classifiers, shows better results than the state of the art, especially when the number of measurements is small. It can reconstruct voxels with 3 crossing fascicles from 10 dMRI measurements, but the method is only tested on synthetic data.
Lightweight CNN ( Z. Lin et al., 2019 )	The model is a CNN that works on cubic patches of size three voxels. It achieves accurate estimation of FOD with 25 measurements. It estimates crossing fibers better than multitissue multishell constrained spherical deconvolution (MSMT-CSD ( Jeurissen et al., 2014 )).
FORDN ( Ye & Prince, 2017 )	This method, named Fiber Orientation Reconstruction guided by a Deep Network (FORDN), is based on overcomplete dictionaries and MLP networks. A coarse dictionary is first used to represent the signal. In the second stage, a larger dictionary is used to compute a finer FOD that is close (in an $\ell_{1}$ sense) to the coarse FOD computed in the first step. Compared with methods that are based on sparse reconstruction or deep learning methods, this method is more accurate especially in voxels with two and three crossing fibers. However, the method is tested on phantom data and in vivo scan of one human subject.
Voxel-wise MLP ( Karimi, Vasung, Jaimes, Machado-Rivas, Warfield, et al., 2021 )	This study uses a voxel-wise MLP that is trained on simulated or real data. Extensive experiments show that this method is superior to a range of conventional FOD estimation methods when the number of measurements is small.
Voxel-wise or small-patch CNNs ( Koppers, Friedrichs, & Merhof, 2017 ; Koppers, Haarburger, Edgar, et al., 2017 )	These studies apply 2D and 3D CNNs on data from individual voxels or small patches to compute the number of fascicles and the complete FOD. Experiments on HCP data show the deep learning methods can estimate the number of major fascicles and the FOD in voxels with complex fiber configurations with as few as 15 measurements and they are more accurate than CSD.
Method for heterogeneous multishell data ( Yao, Newlin, et al., 2023 )	To enable the method to work with different q-space shells, the model has three input heads for three common b-values (1000, 2000, and 3000). Either one of the shells or any combination thereof can be supplied at test time. The architecture itself is a CNN. Only HCP data are used to validate the method.
MLP applied on dMRI signal decay features ( Karimi, Vasung, Jaimes, Machado-Rivas, Khan, et al., 2021 )	A novel feature vector is proposed based on the decay of the diffusion signal as a function of orientation. Using this hand-crafted feature vector as input, an MLP is trained to estimate the “angle to the closest fascicle” for a large set of directions on the sphere. This information is used to infer the number and orientation of the major fascicles or to approximate the complete FOD.
Spherical deconvolution network ( Bartlett et al., 2023 )	The network is inspired by a reformulation of CSD, hence it is presented as a “model-driven deep learning method.” The reformulation is turned into an iterative optimization method that is unfolded and implemented as a deep neural network. The loss function has an $\ell_{2}$ term for the predicted FOD and a cross-entropy term for the predicted number of fixels (i.e., major peaks). The study has used HCP data only.

#### 4.5.1 Method design

Prior to the surge of deep learning, a few studies used classical machine learning methods for FOD estimation (Schultz, 2012;Taquet et al., 2013). One study computed priors from a population of training subjects and employed the learned prior in a maximum a posteriori framework to improve the estimation accuracy on test data (Taquet et al., 2013).

In more recent years, a number of studies have trained neural networks to estimate the FOD (Koppers, Friedrichs, & Merhof, 2017;Nath et al., 2020). These studies are different in terms of the neural network architecture, training strategy, and evaluation metrics. However, most share a similar claim that a deep neural network can estimate FODs more accurately than spherical deconvolution methods. Methodologically, most deep learning studies have followed a straight estimation approach, where the input dMRI measurements are mapped to the target FOD. The input signal and the target FOD are usually expressed either as functions on a discrete spherical grid or using spherical harmonics. However, there have been many exceptions, three of which we briefly describe below.

One study has proposed to use an autoencoder to learn a model of plausible FOD shapes from a high-quality training dataset (Patel et al., 2018). This prior is then used to regularize the CSD method to obtain more accurate predictions than the standard CSD. Results show that this technique outperforms conventional methods when measurements are few or the diffusion strength (i.e., the b-value) is low.Koppers and Merhof (2016)use a succession of two CNN classifiers, where the first CNN estimates the number (either 1, 2, or 3) of fibers in the voxel and the second CNN estimates the fiber orientations.Ye and Prince (2017)used deep neural networks to estimate the FOD by solving sparse estimation problems in dictionaries. A coarse dictionary was used in the first stage to compute an initial estimate, which was then refined using a finer dictionary.Bartlett et al. (2023)start by reformulating the CSD equation to derive an iterative optimization algorithm for FOD estimation. They propose to solve this problem using a deep neural network. In addition to an$\ell_{2}$loss for FOD prediction error, they introduce a cross-entropy loss to encourage correct prediction of the number of major fascicles. Their experiments show that their method performs better than MSMT-CSD in estimating FOD and its peaks.

Unlike the scalar microstructural indices, FOD is a function of angle and, hence, it is rotation sensitive.Elaldi et al. (2021)argue that the standard convolutional layers (which provide equivariance to planar translation) are insufficient for spherical signals in dMRI, where rotation equivariance is additionally needed. Instead, they employ rotation-equivariant graph convolutions proposed byPerraudin et al. (2019). Furthermore, they opt for spherical harmonics of degree 20 (as opposed to degree 6 or 8 in standard methods;Tournier et al., 2007) to enable reconstruction of nearby FOD peaks. They show that, compared with the CSD baseline, their neural network achieves superior FOD estimation and better performance on downstream tasks such as tractography on phantom data and human brain scans. They have further extended this study by developing neural network layers that are equivariant to translations and grid reflections (as are standard FCNs) as well as to voxel and grid rotations (Elaldi et al., 2023). They argue that these extra equivariance properties are needed to give the network the necessary spatiospherical inductive biases to effectively learn to compute the FOD from data. Their results show more accurate FOD estimation, tractography, and brain tissue segmentation in dMRI compared with a range of state-of-the-art methods. Related neural network architectures are reviewed in more detail inSection 5.3.

#### 4.5.2 Training data

The majority of studies have used standard estimation methods such as CSD to generate target FODs for training and validation data (Koppers, Friedrichs, & Merhof, 2017;Yao, Newlin, et al., 2023). Typically, these studies have aimed at matching the standard technique while using fewer measurements as input. It has been claimed that neural networks can use 6–20 measurements from a single shell to estimate the FOD estimated from multishell HARDI data computed with standard methods. Therefore, the validation has been based on comparison with standard methods. This is unlike the common validation approach for FOD estimation techniques, which has been primarily based on simulation (Jelescu et al., 2020). However, with this approach, the trained model will inevitably inherit some of the shortcomings of the standard method that is used to generate the target FOD (Elaldi et al., 2021;K. Schilling et al., 2016;K. G. Schilling et al., 2018). Moreover, the assessments can only tell us how close the new machine learning method is to the standard technique.

A few important studies have used histology data for training and/or validation.Nath, Schilling, et al. (2019)used ground truth histology FOD data from squirrel monkey brains, registered to dMRI data, to assess and compare a deep learning technique and CSD. Their experimental results showed that there was additional information in the dMRI measurements that CSD failed to utilize, and that a deep learning method was capable of using that extra information for FOD estimation. The deep learning model, which was a voxel-wise MLP, outperformed CSD in terms of estimation accuracy using histology as ground truth. Further experiments with in vivo human scans from the HCP dataset showed better scan–rescan reproducibility of the deep learning method, which the authors interpreted as evidence that the method could be used in clinical applications. Other studies (Elaldi et al., 2021,^2023^) have proposed novel methods for sidestepping the need for an FOD ground truth. They compute the convolution of the estimated FOD with the fiber response function to obtain the corresponding dMRI signal and use the difference between this predicted signal and actual measurements as the optimization loss for model training.

#### 4.5.3 Prediction of the number of main fascicles

Instead of estimating the complete FOD, several studies have addressed the less ambitious but still challenging problem of determining the number of major fascicles in each voxel. Schultz trained a support vector regression model to estimate the number of major fascicles using simulated training data (Schultz, 2012). Evaluations on simulated and real brain data showed that this method determined the number of major fascicles more accurately than CSD. Another study (Karimi, Vasung, Jaimes, Machado-Rivas, Khan, et al., 2021) devised novel feature vectors to characterize the decay of the diffusion signal as a function of orientation. The feature vectors were then used by an MLP to compute the angle to the closest fascicle. This information was further processed via smoothing and local minimum extraction to determine the number and orientation of major fascicles in the voxel. Comparisons with several classical methods showed that this machine learning technique was more accurate.

### 4.6 Tractography and structural connectivity

Tractography algorithms build on local fiber orientations to compute virtual streamlines that connect different brain regions (Behrens et al., 2014). Tractography has important applications, most prominently delineation of specific white matter tracts and quantitative structural connectivity (F. Liu et al., 2019;Sotiropoulos & Zalesky, 2019). It is one of the most common, challenging, and controversial computations enabled by dMRI (Lemkaddem et al., 2014;C.-H. Yeh et al., 2021;F. Zhang, Daducci, et al., 2022). Early tractography methods relied on the diffusion tensor model for computing the local fiber orientations (Basser et al., 2000). Over the past two decades, more advanced streamline tracing methods have been developed including anatomically constrained tractography (R. E. Smith et al., 2012), global tractography algorithms (Mangin et al., 2013), ensemble tractography (Takemura et al., 2016), augmented tractography (F.-C. Yeh, 2020), and microstructure-informed tractography filtering (R. Smith et al., 2020). These methods aim to utilize anatomical context information or the correspondence between tractography streamlines and the local dMRI signal to overcome the limitations of conventional tractography techniques.

However, tractography is intrinsically ill-posed and suffers from high false positive and false negative rates (Maier-Hein et al., 2017;Thomas et al., 2014). Some of the main sources of error, unreliability, and ambiguity in tractography include (Rheault, Poulin, et al., 2020;Sotiropoulos & Zalesky, 2019;Yang et al., 2021;F. Zhang, Daducci, et al., 2022) (1) difficulty of modeling crossing fibers, (2) ambiguity of streamline tracing in voxels with crossing or kissing fibers, (3) presence of bottleneck regions, where several tract bundles trace the same voxels in the same direction, and (4) anatomical biases such as the gyral bias and termination bias. Standard tractography methods often produce inaccurate results that can influence tract-specific analysis and structural connectivity assessment (K. G. Schilling, Daducci, et al., 2019;Sotiropoulos & Zalesky, 2019;F. Zhang, Daducci, et al., 2022).

Machine learning may offer a framework for developing better tractography algorithms. They are model free and allow for a seamless integration of anatomical priors and other sources of information that may be useful for tractography. Some of these pieces of information, such as the direction of previous tractography steps, have been used in conventional tractography methods in the past (Lazar et al., 2003;Malcolm et al., 2010). Nonetheless, machine learning methods offer higher flexibility in integrating various inputs in a unified model and to optimize the model to reduce the tractography error with respect to all inputs jointly. As a result, they have the potential to have lower false positive rates, to be more robust to measurement noise, and to overcome the inherent biases of classical tractography algorithms (Neher et al., 2017;Poulin et al., 2019;Sarwar et al., 2020). Although various methods such as self-organizing maps (Duru & Özkan, 2013) and random forests (Neher et al., 2017) have been successfully used for tractography, machine learning-based tractography techniques have increasingly relied on neural networks (Neher et al., 2017;Poulin et al., 2018,^2019^).Table 6lists some of the studies that have developed machine learning methods for streamline tractography. We summarize the main technical aspects of these methods and their reported results below.

**Table 6.** A summary of selected machine learning methods for streamline tractography.

Reference	Model (D: deterministic, P: probabilistic)	Model input	Main findings
Fiber tracking with a random forest classifier ( Neher et al., 2017 )	Random forests (D & P)	Raw diffusion signal in a small neighborhood of the current voxel and direction of the three preceding streamline steps.	Higher sensitivity and specificity and lower angular error than 12 state-of-the-art methods.
Learn to Track ( Poulin et al., 2017 )	GRU (D)	Raw diffusion signal in the current voxel.	Better tract coverage and lower false positive rate than classical methods.
DeepTract ( Benou & Riklin Raviv, 2019 )	RNN (D & P)	Raw diffusion signal in the current voxel.	Lower percentage of invalid connections and nonconnections, higher rate of valid bundles, and higher bundle coverage than a range of learning and classical methods.
Jörgens et al. (2018)	MLP (D)	Raw diffusion signal and directions of the two preceding streamline steps.	This study reports extensive experiments to determine the optimal input and network architectures.
Wegmayr et al. (2018)	MLP (D)	Raw diffusion signal in a $3^{3}$ -voxel neighborhood and directions of the 2 preceding streamline steps.	Better results than standard tractography methods when trained on small datasets. The method is robust to noisy training data.
Cai, Lee, Newlin, Kerley, et al. (2023)	Convolutional-recurrent neural network	Anatomical MRI and streamline memory.	This study has reported that the variability in tractography and structural connectivity metrics computed based on T1-weighted MRI is similar to that for standard dMRI-based results.
W. Liu et al. (2024)	Convolutional, attention, and MLP modules	FOD, brain parcellation, and tissue segmentation maps, prior fixel map and directions of six preceding steps.	This method was designed for *fetal* brain tractography. Experiments showed superior ability to reconstruct various white matter tracts compared with existing methods.
Track-to-Learn ( Théberge et al., 2021 )	Deep reinforcement learning (D & P)	FOD at the current voxel and its six neighbors and directions of the past four streamline steps.	A reinforcement learning approach can be competitive with supervised learning methods while also being more generalizable to unseen or out-of-distribution data.
Bundle-Wise Deep Tracker ( Poulin et al., 2018 )	GRU (D)	dMRI signal.	Superior to classical methods in terms of true positive rate and better bundle volume coverage than existing probabilistic techniques.
Entrack ( Wegmayr & Buhmann, 2021 )	MLP (P)	Directions of the three last streamline steps and FOD, represented as SH of order 4, in a voxel neighborhood of size three voxels.	The method achieves competitive results compared with a range of conventional and machine learning methods on independent synthetic test data.

#### 4.6.1 Method design

An increasingly popular class of models in tractography is recurrent neural networks (RNNs) (Benou & Riklin Raviv, 2019;Cai, Lee, Newlin, Kim, et al., 2023;Jörgens et al., 2018;Poulin et al., 2017,^2018^).Poulin et al. (2017)trained MLPs and Gated Recurrent Units (GRUs) (Cho et al., 2014) to map the dMRI signal to the streamline directions. The input to their model was the b0-normalized signal, resampled to a fixed spherical grid with 100 directions. The information from the preceding tracing steps was passed to the current step via the hidden state vector. Experiments showed that the GRU-based method outperformed 96 competing techniques on a simulated dataset and achieved expert-level results on a scan from the HCP dataset.Benou and Riklin Raviv (2019)used an RNN to estimate a probabilistic FOD, which could then be used for deterministic or probabilistic tractography. The streamline tracing was stopped when the model predicted a termination label or when the entropy of the predicted FOD was below a threshold. This method showed competitive performance compared with classical methods and machine learning techniques, especially in terms of false positive rate and ability to reconstruct different fiber bundles.

The majority of the proposed methods have followed a supervised learning strategy. The main difficulty with these approaches is obtaining rich data with reliable ground truth, discussed further in the next subsection. Given the difficulty of obtaining training data, there is a growing interest in reinforcement learning techniques (Sinzinger & Moreno, 2022;Théberge et al., 2021).Théberge et al. (2021)developed the first deep reinforcement learning-based tractography method. In their method, the state is derived from the diffusion signal while the reward is computed from the FOD peaks and the direction of the streamline. The model input consists of SH-represented FOD at the current voxel and its six neighbors. Additionally, the past four streamline directions are included as extra inputs to the model. The reward function is based on local information. Specifically, it promotes the closeness of the predicted streamline tracing step with the major FOD peaks and encourages streamline smoothness. Experiments show that the reinforcement learning method is competitive with supervised learning methods while also having better generalizability to unseen datasets. However, a true reinforcement learning method requires the knowledge of streamline start and end points, which is unavailable for in vivo data.

Designing a machine learning-based tractography method involves several important choices including (1) model input, (2) whether to adopt a probabilistic or a deterministic streamline propagation approach, (3) classification (Jörgens et al., 2018;Neher et al., 2015) or regression (Poulin et al., 2017;Wegmayr & Buhmann, 2021) strategy, and (4) bundle-specific (Poulin et al., 2018;Reisert et al., 2018) or whole-brain tractography (Benou & Riklin Raviv, 2019;Poulin et al., 2017). Most studies have not justified their choices and there is a lack of experimental results to inform these decisions.

#### 4.6.2 Training data

One approach to obtaining ground truth tractography data is via elaborate manual editing of automatically generated tractograms by experts (Maier-Hein et al., 2016). However, even for experts, it is often impossible to resolve ambiguous situations, and this practice suffers from high interobserver variability (Rheault, De Benedictis, et al., 2020;K. G. Schilling et al., 2021a). Physical phantoms represent an alternative (Neher et al., 2014;Wilkins et al., 2012).Poupon et al. (2010;Fillard et al., 2011) have developed the FiberCup phantom to simulate a coronal slice of the brain at the level of corticospinal tracts. This phantom includes a rich set of crossing, fanning, and kissing tracts as well as U-fibers. The main problem with this approach is that no phantom can represent the complexity and intersubject variability of the human brain. Lack of reliable data for model development and validation has remained one of the main stumbling blocks for advancing machine learning-based tractography, similar to other applications discussed in this paper. A notable recent database that has been made publicly available is TractoInferno (Poulin et al., 2022). It includes multiscanner data acquired with different protocols. In addition to the processed dMRI data, TractoInferno includes 30 white matter bundles reconstructed using an ensemble of 4 different tractography approaches followed by automatic and manual quality control.

#### 4.6.3 Evaluations and main results

Machine learning methods have been effective in reconstructing various tracts with low false positive rates, reducing the systematic errors and biases that are prevalent with classical tractography methods, and improving generalizability to unseen data (Neher et al., 2015;Poulin et al., 2019). Several reasons have been put forward to explain the success of machine learning methods. First, they avoid making rigid, ad-hoc, and possibly suboptimal modeling assumptions or streamline propagation and stopping rules that are common in standard methods (Neher et al., 2015;Poulin et al., 2019;Wegmayr & Buhmann, 2021). Some of the proposed machine learning methods use the dMRI data (rather than diffusion tensor or FOD estimates) as input, thereby sidestepping the unavoidable errors in fiber orientation estimation (Benou & Riklin Raviv, 2019;Neher et al., 2015;Wegmayr et al., 2018). Moreover, they have the potential to learn tissue probabilities from dMRI data, rather than relying on the information provided by a registered anatomical MRI or ambiguous parameters such as FA. Machine learning methods can also learn the noise and artifacts from data, instead of imposing simplified noise models and assuming artifact-free scans (Neher et al., 2015). Furthermore, these methods can incorporate estimation uncertainty into streamline tractography in a systematic way (Wegmayr & Buhmann, 2021). Machine learning techniques are also by far more flexible in incorporating nonlocal information, such as anatomical context, which can greatly improve tractography results (Poulin et al., 2019). An important piece of information that can be easily incorporated into most machine learning models is the directions of preceding streamline tracing steps. The direction of the last step indicates the streamline slope and the directions of the last two steps indicate its curvature. This information has been useful in improving tractography accuracy (Poulin et al., 2017). Incorporation of the direction of preceding steps can also improve the tractography in voxels with crossing fibers and bottleneck regions (Poulin et al., 2017;Théberge et al., 2021).

#### 4.6.4 Tractography postprocessing

Machine learning methods have also been used to enhance the generated tractograms via postprocessing operations such as identification of false positives or anatomically implausible streamlines. Deep learning has shown great success in this task as well. An example of a supervised method is the study ofAstolfi et al. (2020), where an existing method (Petit et al., 2019) is used to generate labels on training data based on anatomical priors. A limitation of supervised approaches is that it is hard to obtain training data that can adequately represent the complete range of invalid streamlines (Ugurlu et al., 2019). Unsupervised methods sidestep this limitation. An example of unsupervised techniques is the autoencoder method proposed byLegarreta et al. (2021). This method uses unlabeled whole-brain tractograms to learn a low-dimensional representation of valid streamlines, which can then be used to detect spurious or anatomically invalid streamlines in the test data. Both supervised and unsupervised approaches have reported remarkable success. A generative method based on autoencoders has recently been proposed to improve the streamline density for tracts that are hard to trace with conventional propagation methods (Legarreta et al., 2023). Using anatomically valid training streamlines, it learns a latent space representation that can be subsequently used as a “streamline yard” to synthesize new streamlines for sparsely populated tracts in a test sample. The synthesized streamlines are accepted or rejected based on a set of criteria related to streamline geometry, connectivity, and agreement with local fiber orientation directions. Experiments with phantom and real data showed that this method significantly improved tract coverage.

### 4.7 Delineation of white matter tracts

The brain white matter is organized into distinct tracts, which consist of bundles of myelinated axons that connect different brain regions such as the cerebral cortex and the deep gray matter (Bullock et al., 2022;Wakana et al., 2004;Wycoco et al., 2013). Although they are tightly packed and often cross one another, each tract has a different function and connects different regions of the brain. Accurate segmentation of these tracts is needed in clinical studies and medical research. For example, in surgical planning, one needs to know the precise extent of the individual tracts in order to assess the risk of damage to specific neurocognitive functions that may result from surgical removal of brain tissue (Kamada et al., 2005;Yang et al., 2021). Furthermore, changes in the tissue microstructure on specific tracts can be associated with brain development and disorders (Bubb et al., 2018;Glasser & Rilling, 2008;Zhuang et al., 2010).

Diffusion MRI is the only noninvasive method that can accurately delineate white matter tracts (Wycoco et al., 2013). Individual tracts may be extracted from whole-brain tractograms by specifying inclusion and exclusion regions of interest (ROIs). This process, which is usually referred to as “virtual dissection,” is time consuming, requires substantial expertise, and suffers from high interobserver variability and low reproducibility (K. G. Schilling et al., 2021a;Wakana et al., 2007).

Several prior studies have aimed at automating the virtual dissection process by learning to compute the inclusion/exclusion ROIs (Suarez et al., 2012;Warrington et al., 2020;Yendiki et al., 2011). Moreover, there has been much effort to develop fully automatic methods for tract delineation. These methods can be divided into two categories: (1) methods that start by performing streamline tractography and (2) methods that do not depend on tractography. We have summarized some of the studies in these two classes of methods, respectively, inTables 7and8. Given the large number of published methods, these tables only list a selection of more recent methods with a focus on deep learning techniques. Below, we describe our main findings from our study of these studies.

**Table 7.** A summary of selected tractography-based methods for automatic delineation/segmentation of white matter tracts.

Reference	Model	Input	Number of tracts	Methodology and results
Deep white matter analysis (DeepWMA) ( F. Zhang et al., 2020 )	CNN	Tractogram	54	The method is based on hand-crafted features describing the streamline geometry. A CNN is applied to classify the streamlines based on the feature vectors. The method shows high accuracy across the human lifespan from neonatal to old age and it works well on brains with gross pathologies such as tumors.
DeepBundle ( F. Liu et al., 2019 )	Graph CNN	Tractogram	12	A graph CNN is used to classify streamlines based on their geometry. Only HCP data are used in this study. The method shows higher accuracy than standard methods, especially for small tracts such as fornix and commissure anterior.
BrainSegNet ( T. Gupta et al., 2017 )	Bidirectional LSTM	Tractogram	8	The method works in two steps, in the first step classifying the white matter vs. the gray matter, and in the second step segmenting the tracts. The method achieves streamline classification accuracy of >96% and recall of >73% , but it is tested on three subjects only.
FiberNET ( V. Gupta et al., 2018 )	CNN	Tractogram	17	The method uses a harmonic function to compute level-sets based on a brain shape-center, thereby reparameterizing the streamlines in a manner that is consistent across subjects. A CNN classifies the streamlines based on this representation. The method achieves low false positive rates.
H. Xu et al. (2019)	CNN	Tractogram	64	The focus of this study was on detecting eloquent white matter tracts for epilepsy surgery. Different CNN architectures and loss functions were investigated. The models were tested on healthy children and children with epilepsy. A deep CNN with attention mechanism achieved the highest F1 score of 0.95.
Spectral clustering ( O’Donnell & Westin, 2007 )	Spectral embedding clustering	Tractogram	10	The method achieves high tract segmentation accuracy and can be useful for cross-subject white matter studies.
Dayan et al. (2018)	Restricted Boltzmann machines (RBM)	Tractogram	61	The method offers memory-efficient unsupervised detection of anatomically relevant tracts without the need to explicitly define the tracts. It achieves Dice Similarity Coefficients ranging from under 0.30 to above 0.90 on different tracts.
TRACULA ( Yendiki et al., 2011 )	Bayesian model	Tractogram and anatomical MRI	18	The model trained on healthy subjects worked well on schizophrenia patients.
FS2Net ( Jha et al., 2019 )	LSTM	Tractogram	8	The authors claim that, because their method relies on streamline geometry, it is particularly useful in situations where the test brains are arbitrarily oriented. They report accuracies above 0.90 but only on three subjects.
Deep Fiber Clustering ( Y. Chen et al., 2023 )	Siamese Graph convolutional neural network	Tractogram and gray matter parcellation	Unclear	The method produces accurate delineation of tracts across age and gender for healthy as well as diseased brains.
Superficial White Matter Analysis (SupWMA) ( Xue, Zhang, et al., 2023 )	MLP	Tractogram	198	This method is based on a point cloud-based representation of the streamlines and contrastive supervised learning. Experimental results on six datasets across the human lifespan show that this approach can accurately extract superficial white matter tracts.
Ugurlu et al. (2019)	Ensemble of MLPs	Tractogram and FOD map	9	This paper presents a way of synthesizing a rich set of invalid streamlines to enable effective training of the model to reject such streamlines in test data. The method is only validated on HCP data.
AnatomiCuts ( Siless et al., 2018 )	Hierarchical clustering	Tractogram and cortical and subcortical segmentation	18	This method advocates for leveraging the position of the streamline with respect to cortical and subcortical landmarks. Experiments on HCP data show that this approach improves the overlap between automatic and manual tracts by 20%.
TractCloud ( Xue, Chen, et al., 2023 )	MLP and Point cloud-based networks	Tractogram	42	In addition to the streamline to be classified, 20 nearest neighbors and 500 streamlines randomly selected from the tractogram are used as input to the MLP. The latent representation computed by the MLP is used by the point cloud network to classify the streamline. The method is validated on five datasets.
TRACULInA ( Zöllei et al., 2019 )	Bayesian model	Tractogram and anatomical MRI	14	This model addresses the neonatal age range and is in part based on TRACULA ( Yendiki et al., 2011 ). It is successfully validated on out-of-distribution data such as prematurely born neonates.

**Table 8.** A summary of selected nontractography-based methods for automatic delineation/segmentation of white matter tracts.

Reference	Model	Input	Number of tracts	Methodology and results
TractSeg ( Wasserthal, Neher, & Maier-Hein, 2018 )	Set of 2D CNNs	Orientation of the major FOD peaks	72	TractSeg was the first study to demonstrably show that deep learning models can segment various white matter tracts without the need for tractography. It achieved an average Dice Similarity Coefficient of 0.84 on HCP data and 0.82 on clinical quality data.
W. Liu et al. (2022)	CNN	Orientation of the major FOD peaks	72	This study showed that modeling the label correlations improved the segmentation accuracy, especially for tracts that were more difficult to segment. Experiments show that this method works better than an atlas-based method and TractSeg ( Wasserthal, Neher, & Maier-Hein, 2018 ).
Lu et al. (2021)	CNN	Orientation of the major FOD peaks	72	This study advocates for self-supervised pretraining of the CNN using two pretext tasks: (1) tractography density prediction, (2) segmentation with noisy labels generated with an atlas-based method. It reports improved segmentation accuracy.
Lucena et al., (2022 , 2023)	CNN	dMRI data represented in a spherical harmonics basis	72	This study evaluates the accuracy of the dMRI-based segmentation in terms of its overlap with the responses of navigated transcranial magnetic stimulation.
Kebiri et al. (2023)	CNN	dMRI data	72	This study showed that it was possible to segment all 72 tracts from the TractSeg study using only six dMRI measurements as input. However, only one independent dataset was used for validation.
HAMLET ( Reisert et al., 2018 )	CNNs with rotation equivariance	Raw diffusion data	12	The CNN model uses rotationally equivariant convolutional operations, thereby eliminating the need to learn data orientations. Experiments show high test–retest reproducibility and generalizability on low-quality data from an external scanner.
Neuro4Neuro ( B. Li et al., 2020 )	CNN	Diffusion tensor	25	This method produced highly accurate and reproducible tract segmentations and it was generalizable to unseen data from external scanners and from dementia patients.
H. Xu et al. (2023)	CNN	Orientation of the major FOD peaks	72	This study used a registration-based label propagation method to synthesize noisy labels for unlabeled images, thereby increasing the size of labeled training data. The authors reported that with this approach, only a single manually labeled image was sufficient for training.

#### 4.7.1 Methods that rely on tractography

A large class of automatic tract delineation methods is based on processing of whole-brain tractograms (Clayden et al., 2007;Garyfallidis et al., 2018;Labra et al., 2017;Legarreta et al., 2021;Siless et al., 2018,^2020^). There are two common approaches used by these methods.

1. One set of methods compare individual streamlines with a predefined set of fibers in an atlas and assign each streamline to a specific tract in the atlas (Garyfallidis et al., 2018;Labra et al., 2017;Wassermann et al., 2010).
2. Another set of methods cluster the streamlines based on some measure of pair-wise similarity (Garyfallidis et al., 2012;O’Donnell & Westin, 2007). There exist various techniques for implementing the clustering, for example, using normalized cuts (Brun et al., 2004) or k-nearest neighbors (J. Wang & Shi, 2019).

Both these subclasses of methods can be computationally expensive, especially for larger number of streamlines and higher image resolution. Consequently, much effort has been directed toward developing fast streamline clustering methods (Garyfallidis et al., 2012;Vázquez, López-López, Sánchez, et al., 2020;J. Wang & Shi, 2018). Some techniques additionally take into account the location of the streamlines relative to anatomical landmarks in the brain (H. Li et al., 2010;Maddah et al., 2008;Siless et al., 2018,^2020^). Most clustering-based methods aim at finding streamlines that have similar geometric shapes and spatial extents. There are also methods that cluster streamlines based on their start/end points (Wassermann et al., 2016). However, methods that use streamline start/end points may not perform as well as streamline clustering techniques (F. Zhang et al., 2017).

Overall, methods that use anatomical information achieve better results than methods that rely on streamline shape alone. Such information can be encoded and incorporated in the tract segmentation computation in various ways. For example,Maddah et al. (2008)used the anatomical information provided by an atlas of white matter tracts, whileTunç et al. (2014)andTunç et al. (2016)used the connectivity signature of the streamlines.Yendiki et al. (2011)have proposed a method that uses the tractography results computed from the dMRI signal and the anatomical tissue segmentation information for automatic tract delineation. Theirs is a probabilistic method, where prior probabilities of the tracts are computed from a set of manually segmented training subjects. This method, trained with data from healthy subjects, performed well on schizophrenia patients. It has been extended to neonatal brain using a dedicated image processing pipeline to address the challenges of early brain data (Zöllei et al., 2019). This extended method also showed good generalizability to data from term- and preterm-born neonates imaged with different scanners and acquisition protocols.

Sydnor et al. (2018)critically compared a manual ROI-based method (Catani & De Schotten, 2008) and two automatic tractography-based tract segmentation techniques (O’Donnell & Westin, 2007;Wassermann et al., 2016) in terms of various measures of accuracy and reproducibility. The results showed that although clustering methods were overall better, they were not consistently superior and that an optimal method might have to rely on a combination of the two approaches. In a rather similar work,O’Donnell et al. (2013)compared streamline clustering methods with techniques that clustered streamlines based on their start and end points in the cortex. They highlighted the pros and cons of each class of methods and advocated for hybrid methods that could benefit from the strengths of both classes of techniques. Similarly,Chekir et al. (2014)andQ. Xu et al. (2013)point out the limitations of the purely clustering-based methods and methods that are based on anatomical priors. They propose methods that synergistically combine the advantages of the two strategies.

A growing number or studies have applied deep learning methods on whole-brain tractograms to extract/cluster individual tracts. These methods have followed very different designs. Given the high flexibility in designing deep learning methods, some of them cannot be categorized under any of the two broad classes mentioned above. A typical recent example is Deep White Matter Analysis (DeepWMA;F. Zhang et al., 2020). DeepWMA first computes a set of feature descriptors to represent the spatial coordinate location of points along each streamline. In order to render the feature descriptor independent of the orientation/order of streamline, the coordinates are also flipped and added to the descriptor via concatenation. The resulting 1D representation is then repeated row wise to create a 2D feature map that a CNN can efficiently process. A CNN is trained to classify the streamlines based on the feature maps. In order to enable their model to identify false positive streamlines, they include an extra class label to represent those streamlines. In DeepBundle (F. Liu et al., 2019), in contrast, streamlines are represented in terms of the point coordinates and a graph CNN is trained to extract geometric features to classify the streamlines. Hence, DeepBundle sidesteps the manual feature engineering that is used in some tractography-based methods. The graph CNN includes a series of graph convolution and graph pooling layers. TRAFIC (Lam et al., 2018) computes streamline curvature, torsion, and Euclidean distance to anatomical landmarks for each point on the streamline. Fibernet (V. Gupta et al., 2017,^2018^), in contrast, represents the streamlines using an isosurface map of the brain. At least one study has observed that using streamline coordinates as input leads to more accurate tract segmentation than features such as curvature and torsion (H. Xu et al., 2019). TractCloud (Xue, Chen, et al., 2023) uses the streamline to be classified, its nearest neighbors, and a set of randomly selected streamlines from the whole-brain tractogram as input to an MLP to compute a latent representation, which is then used by a point cloud network to classify the streamline.

#### 4.7.2 Methods that avoid tractography

In addition to the streamline similarity measures and anatomical information described above, tractography-based methods have resorted to various other information to improve their accuracy. These include homology between the brain hemispheres, spatial and shape priors, and population averages (atlases). However, all tractography-based methods are inherently limited by the errors in streamline tracing (Hua et al., 2008;Maier-Hein et al., 2017). Moreover, they typically involve several processing steps such as streamline propagation, white matter segmentation, gray matter parcellation, and clustering or similar computations to extract individual tracts. Each of these computations may introduce its own errors that depend on method settings, and it is difficult to jointly optimize all these processing steps. Moreover, some of these computations, for example, tractography and streamline clustering, can require long computation times.

To avoid these errors and limitations, several studies have proposed to segment the tracts on diffusion tensor or fiber orientation images, thereby avoiding the tractography. Some of the classical machine learning methods that have been used for this purpose include Markov Random Fields (Bazin et al., 2011), k-nearest neighbors technique (Ratnarajah & Qiu, 2014), level-set methods (W. Guo et al., 2008;Jonasson et al., 2005;Lenglet et al., 2006), and template/atlas-based techniques (Eckstein et al., 2009;Hua et al., 2008).

More recently, deep learning has shown unprecedented accuracy for this application as well (Dong et al., 2019;Wasserthal, Neher, & Maier-Hein, 2018).Wasserthal, Neher, and Maier-Hein (2018)represented overlapping/crossing tracts using a simple but powerful concept that they named tract orientation maps (TOMs). Each tract is represented by a separate TOM, where each voxel contains a vector representing the local orientation of that tract. If a tract does not cross a certain voxel, the value of that voxel in the TOM for that tract will be a vector of zeros. In their framework, a standard method such as CSD is applied to compute the FOD, and the FOD peaks are extracted to build the TOMs. These peak orientations are fed into an FCN, which is trained to compute the TOMs. The TOMs can be used as a prior or direct input for bundle-specific tractography. The authors claim that their method is a viable solution to excessive false positive rate of standard tractography techniques. They support this claim by demonstrating that their method leads to superior tract bundle reconstruction compared with several state-of-the-art methods such as TractQuerier (Wassermann et al., 2016), RecoBundles (Garyfallidis et al., 2018), and WhiteMatterAnalysis (WMA) (O’Donnell & Westin, 2007). In subsequent studies, they build upon this method and extend it in a few important directions. In TractSeg (Wasserthal, Neher, & Maier-Hein, 2018), they develop an FCN-based method to map the TOMs to the segmentation probability maps for each tract. To enable the method to run on limited GPU memory while working on full-resolution images to segment the complete set of 72 tracts, their model is a 2D FCN that works on axial, coronal, and sagittal slices separately. TractSeg achieves a mean Dice Similarity Coefficient (DSC) of 0.84. On all tracts except for commissure anterior and fornix, it achieves a mean DSC of higher than 0.75. While the tract segmentation masks generated by TractSeg can be used for accurate bundle-specific tractography (Wasserthal, Neher, & Maier-Hein, 2018), a more complete method is presented in a subsequent study by the same authors (Wasserthal et al., 2019), where a separate deep learning model segments the start and end regions of each tract.

A number of studies have built upon the methods proposed byWasserthal, Neher, and Maier-Hein (2018)andWasserthal et al. (2019). A representation similar to TOM was proposed in HAMLET (Reisert et al., 2018), where the model output is a tensor field that indicates the presence and local orientation of a tract.B. Li et al. (2020)trained a CNN to segment 25 white matter tracts directly from diffusion tensor images. Their extensive experiments showed high segmentation accuracy, low scan–rescan variability in tract-specific FA analysis, good generalizability to cross-center data, and successful application in a tract-specific population study on dementia patients. Their method was more accurate than tractography-based techniques, while being orders of magnitude faster. They also found that adding anatomical (T1-weighted) images to the diffusion tensor as input did not improve the segmentation accuracy. Moreover, using the directions of the three major peaks led to lower accuracy than diffusion tensor (B. Li et al., 2020).W. Liu et al. (2022)followed an approach similar to TractSeg but proposed to model the correlation between tract labels. Specifically, they introduced an auxiliary neural network that mapped the native tract labels into a lower dimensional label space. The main segmentation network predicted this lower dimensional label, which could be used to predict the tract segmentation maps. The authors argue that this approach makes the task of tract segmentation simpler to learn and show that it can improve the segmentation of certain tracts such as the fornix that are especially difficult to segment with TractSeg. Similarly,Lu et al. (2021)follow the general approach proposed by TractSeg, but propose self-supervised pretraining and transfer learning (Lu & Ye, 2021) to reduce the required labeled data. These studies have shown that as few as five labeled images may be sufficient to train CNNs for segmenting certain tracts. To reduce the required manual labeling, another study proposed a deep learning registration method to align the labeled images to unlabeled images, thereby synthesizing a large training dataset (H. Xu et al., 2023). Experiments showed that even a single labeled image was sufficient to train an accurate deep learning model.

In order to determine the orientation of major fascicles, previous studies have relied on computation of the diffusion tensor or FOD. However, none of these intermediate computations have an unambiguous biophysical meaning and they entail unavoidable estimation errors. Moreover, the intermediate computations for most existing methods assume availability of dense multishell measurements, which are not acquired in many clinical and research applications. Recently, some studies have suggested to perform the segmentation based directly on the dMRI signal (Kebiri et al., 2023;Lucena et al., 2022,^2023^;Mukherjee et al., 2020). One study demonstrated the feasibility of segmenting the corticospinal tract (CST) from the dMRI data for neonatal subjects in the dHCP dataset (Mukherjee et al., 2020). Another study segmented the 72 tracts from the TractSeg study (Kebiri et al., 2023). It showed that it was possible to achieve a similar level of segmentation accuracy as TractSeg while using only six measurements.

#### 4.7.3 Superficial white matter analysis methods

While most studies have focused on deep white matter, a few studies have addressed the arguably more challenging task of segmenting the superficial white matter tracts, also known as the U-fibers (P. Guevara et al., 2012;Román et al., 2017;Vázquez, López-López, Houenou, et al., 2020;Xue, Zhang, et al., 2023). These tracts are critical for assessing brain connectivity as they account for the majority of the corticocortical connections. However, they are difficult to study with standard tractography techniques due to their small size, partial volume effects, and high intersubject variability (M. Guevara et al., 2020;Román et al., 2017;Vázquez, López-López, Houenou, et al., 2020).Xue, Zhang, et al. (2023)developed a neural network model to segment 198 superficial white matter tracts via clustering the streamlines using a deep neural network. Streamlines were represented as point clouds and a contrastive learning approach was used to train the network. The authors reported remarkable accuracy (cluster identification rates of 87–99%, except for a neonatal dataset) for different age groups and health conditions including brain tumor patients. Another study (Román et al., 2017) developed a hierarchical clustering method to identify representative groups of short association fibers from a population of subjects. This was accomplished by identifying fibers that were present in the majority of the subjects in a common (Talairach) space. The method was used to develop an atlas of 93 reproducible tracts, which could then be used to segment these tracts from whole-brain tractograms of test subjects. Another study suggested clustering of superficial white matter tracts based on their connectivity patterns using cortical brain parcellations inferred with an atlas (Vázquez, López-López, Houenou, et al., 2020).

### 4.8 dMRI registration

Tract-specific studies, as their name implies, study the organization, development, and tissue microstructure of specific white matter tracts (S. M. Smith et al., 2014). They can be of two types: (i) longitudinal studies consider the same subject(s) over time, while (ii) population studies compare different cohorts of subjects such as diseased versus control groups. They are among the most common dMRI studies because many neurodevelopmental deficits and neurological disorders are linked to damage to the tissue microstructure on specific tracts (Pini et al., 2016;Sexton et al., 2011). The success of these studies depends on ensuring that the same tracts are compared between scans/subjects, which is typically achieved via precise alignment of different brain scans. Regardless of the approach taken, this is a complex and error-prone computation. Existing computational methods vary greatly in terms of spatial accuracy and the required manual input (S. M. Smith et al., 2014). Voxel-based morphometry methods are simple but incapable of accurate tract-specific comparisons (Ashburner & Friston, 2000), while semiautomatic and automatic tractography-based methods are limited by the tractography errors (Pagani et al., 2005;Suarez et al., 2012). Among the automatic methods, TBSS (S. M. Smith et al., 2006) is the most widely used. However, it suffers from important inaccuracies and shortcomings (Bach et al., 2014;Edden & Jones, 2011;Madhyastha et al., 2014). Machine learning techniques have a unique potential to develop more reliable methods to address these limitations.

A main source of error in many tract-specific analysis methods is poor registration. Some methods, such as TBSS, perform the registration based on FA images, which ignores the orientation-dependent microstructure information. Leveraging the fiber orientation information may significantly improve alignment accuracy (Raffelt et al., 2011;H. Zhang et al., 2006). Several deep learning-based dMRI registration methods have been proposed in the past few years and they have shown a tremendous potential for accurate and robust registration (Bouza et al., 2023;Grigorescu et al., 2020,^2022^;F. Zhang, Wells, & O’Donnell, 2021). Overall, these methods follow the recent advancements in deep learning-based registration, which are mainly based on fully convolutional networks, spatial transformer networks, and unsupervised training (Balakrishnan et al., 2019). Additionally, they usually use the finite strain technique (Alexander et al., 2001) to properly reorient the white matter structures based on the computed nonlinear deformations. One study has shown that using the diffusion tensor images in addition to anatomical (T1- and T2-weighted) images can lead to higher registration accuracy (Grigorescu et al., 2020). A subsequent study has shown that attention maps can be learned to weight the contribution of the two modalities (i.e., anatomical and microstructural images) to achieve better registration (Grigorescu et al., 2022). Another study used the tract orientation maps (Wasserthal, Neher, & Maier-Hein, 2018) for 40 white matter tracts as well as FA maps to compute 41 separate deformation fields (F. Zhang, Wells, & O’Donnell, 2021). A fusion neural network combined these into a final deformation field. On data from different age groups, scanners, and imaging protocols, this method was more accurate than existing DTI- and FOD-based registration techniques.

### 4.9 Tract-specific analysis

A few studies have proposed machine learning methods for cross-subject tract-specific analysis. Most of these methods, but not all, are based on tract segmentation and alignment of data from different subjects/scans. Such methods are urgently needed since existing solutions such as TBSS are known to suffer from important limitations. These studies are briefly discussed below under two classes of approaches.

#### 4.9.1 Tractography-based methods

Prasad et al. (2014)developed a framework that consisted of streamline clustering, skeletonization based on highest streamline density, and registration based on geodesic curve matching. They showed that this method had a higher sensitivity than TBSS.Jin et al. (2014)proposed a similar approach based on multiatlas clustering of streamlines with label fusion.F. Zhang et al. (2018)used group-wise registration and analysis of whole-brain tractograms to compute an atlas of valid streamlines, which they then used to extract subject-specific fiber tracts. They used this method to analyze the tissue microstructure in terms of FA and MD for a population of autism spectrum disorder children and healthy controls. If the goal is to predict subject-level neurocognitive scores, cross-subject data alignment or spatial correspondence can be side-stepped. TractGeoNet (Y. Chen et al., 2024), for example, uses a neural network to directly predict the score from streamline-level tissue microstructure information.

#### 4.9.2 Deep learning-based methods

Segis-Net (B. Li, Niessen, Klein, de Groot, et al., 2021) is a deep learning method for tract segmentation and registration. The segmentation and registration modules are optimized jointly. The segmentation module uses diffusion tensor as input, while the registration module uses FA images as input. The loss function includes separate terms for segmentation and registration as well as a joint term to encourage tract segmentation overlap between pairs of registered images. The advantage of this approach is the joint learning of the segmentation and registration tasks and reliance on an implicit unbiased reference image (B. Li, Niessen, Klein, Ikram, et al., 2021). Segis-Net was applied to analyze six tracts in a longitudinal study including 8045 scans and was shown to lead to higher reproducibility than a nonlearning method and a machine learning pipeline that addressed segmentation and registration tasks separately. Segis-Net also reduced the sample size requirements for tract-specific studies by a factor of up to three and it was orders of magnitude faster than methods based on conventional segmentation and registration techniques. In another study, the authors develop an accurate and detailed dMRI atlas that includes 72 tracts (Karimi et al., 2023). For each test subject, the method segments the tracts and registers the subject data onto the atlas. This method offers higher reproducibility and better robustness to measurement noise than TBSS. In a different study, an autoencoder has been proposed to detect abnormal tissue microstructure indices on white matter tracts (Chamberland et al., 2021). The autoencoder input consists of microstructural indices on white matter tract profiles. Following the standard autoencoder approach, the model is trained on a population of normal subjects to learn to reconstruct healthy brain data. When applied on data from pathological brains, the autoencoder can detect abnormal microstructure indices via large reconstruction errors. The authors validate this method on data from patients with various neurological disorders including epilepsy and schizophrenia. These experiments show that the new method is more accurate than standard abnormality detection techniques. The authors characterize their method as moving beyond group-wise analysis and enabling assessment of single patients at an individual level. However, one can argue that techniques such asKarimi et al. (2023)andB. Li, Niessen, Klein, de Groot, et al. (2021)can offer the same capability.

### 4.10 Segmentation

Tissue segmentation in the dMRI space is needed for anatomically constrained tractography and structural connectivity analysis (R. E. Smith et al., 2012;Sotiropoulos & Zalesky, 2019). Given the lower spatial resolution and tissue contrast of dMRI, this segmentation is usually obtained via segmenting anatomical MRI data followed by registration to the dMRI space. This registration can introduce significant error because the contrast and image distortions between the modalities can be very different. Therefore, direct tissue segmentation in dMRI is desirable. Machine learning methods, and in particular deep learning techniques, have shown great promise here as well.

Prior to the development of deep learning-based segmentation methods, classical machine learning methods such as fuzzy c-means clustering with spatial constraints (Wen et al., 2013) and sparse representation of the dMRI signal in dictionaries (Yap et al., 2015) were used to segment the brain tissue.Ciritsis et al. (2018)determined the optimal set of dMRI measurements and DTI features for tissue classification with support vector machines (SVM).Schnell et al. (2009)computed rotation-invariant features from HARDI measurements and used them as input to an SVM to segment the brain tissue into six classes. The class labels included white matter with parallel (single) fibers and white matter with crossing fibers.

More recently,Golkov et al. (2016)extended their deep learning tissue microstructure estimation technique to develop a segmentation method that mapped the dMRI signal in each voxel to a probability for white matter (WM), gray mater (GM), cerebrospinal fluid (CSF), and MS lesion. Their simple and flexible approach allowed them to include an extra FLAIR channel, which could be especially useful for MS lesion segmentation. Their model accurately segmented the brain tissue, and detected MS lesions with area under the receiver operator characteristic curve (AUC) in the range 0.88–0.94. The same authors also proposed a variational autoencoder model for novelty detection based on q-space dMRI data and showed that it could accurately segment MS lesions with an AUC of approximately 0.90 (Vasilev et al., 2020). Another study used DTI and DKI features as input to a multiview CNN to achieve accurate segmentation of WM, GM, and CSF (F. Zhang, Breger, et al., 2021). The model was trained on HCP-quality data, where the anatomical and dMRI data could be registered precisely. The trained model achieved high segmentation accuracy on clinical dMRI acquisitions. The same research team trained a deep learning model to parcellate the brain into 101 regions using DTI-derived parameter maps as input (F. Zhang et al., 2023). The authors showed the utility of the new method in improving tract-specific streamline bundle extraction from whole-brain tractograms.

A recent study addressed the challenging task of*fetal*brain segmentation in dMRI (Calixto, Jaimes, et al., 2024). This is a particularly difficult application due to the low contrast of fetal brain dMRI. The authors used DTI-derived parameters as the input to their model, which was based on vision transformers. They proposed a novel self-supervised learning approach that enabled them to train the model on a large unlabeled dataset and a smaller labeled dataset. This method achieved a mean DSC of 0.84–0.90 on different tissue types. Experiments showed that this method drastically improved fetal brain tractography. Building upon their improved tractography results, the same research team has developed a multitask learning method that segments the brain tissue, white matter tracts, and gray matter regions (Karimi et al., 2024). For isointense stage of brain development where white matter and gray matter have similar intensity in T1- and T2-weighted images (approximately between 6 and 8 months), one study used an additional FA channel to accurately segment the brain tissue with an FCN (W. Zhang et al., 2015). Segmentation of the intracranial volume also known as brain masking or skull stripping, which is typically performed as a preprocessing step, has also been addressed with machine learning methods (Faghihpirayesh et al., 2023;Karimi, Dou, et al., 2020;Reid et al., 2018;X. Wang et al., 2021).

## 5 Discussion

This section presents some thoughts on the application of machine learning for dMRI analysis. It discusses important factors that need to be taken into considerations when designing, training, and validating new methods. Additionally, it points out some of the limitations of prior studies, persistent challenges, and topics that require further investigation.Figure 2shows the outline of the topics discussed in this section.

### 5.1 Validation

A shortcoming of many of the studies discussed above is with regard to validation. This subsection breaks down the main aspects of this limitation.

#### 5.1.1 Validation on abnormal/pathological data

The methods are usually trained and validated on data from normal brains. This can be a major flaw because brain pathologies may represent vastly different and heterogeneous structure and tissue microstructure that drastically alter the dMRI signal (Jelescu et al., 2020;Reynaud, 2017;Szczepankiewicz et al., 2016). Brain pathologies such as tumors can also change the brain anatomy and impact the performance of automatic tract analysis methods (Lucena et al., 2022,^2023^). For tract delineation, one study has shown that on pathological brains conventional atlas-based methods work better than the more recent deep learning techniques (Young et al., 2024). Another study (Peretzke et al., 2023) found that the accuracy of automatic tract segmentation methods such as TractSeg dropped significantly (DSC = 0.34) on brains with tumors. A new human-in-the-loop method based on active learning achieved a mean DSC of 0.71 on the same dataset.

For tissue microstructure mapping, some studies have validated the new methods on selected pathologies such as MS (Golkov et al., 2016) and stroke (H. Li et al., 2021). One study trained a model on healthy brain data to jointly estimate the DTI, DKI, and multicompartment models and tested the model on stroke patients (HashemizadehKolowri et al., 2022). Results showed that the model accuracy on pathological tissue suffered more for multicompartment models (e.g., NODDI) than for DTI. Similarly, a few studies have validated tract-specific analysis methods on clinical data. For example, one study (Tallus et al., 2023) showed that automatic tract segmentation with TractSeg (Wasserthal, Neher, & Maier-Hein, 2018) was more accurate in detecting microstructural changes due to traumatic brain injury than a method based on manual tract segmentation.B. Li et al. (2020)validated their joint tract segmentation and registration method on a clinical population. Although most of the published results on abnormal brains are positive and encouraging, they are not sufficient. Validation of deep learning methods on abnormal brain data is especially important because these methods are highly complex and difficult to interpret, and because the generalizability of these methods to unseen data is hard to predict (Arrieta et al., 2020). As an example, for tissue microstructure mapping, one study has found that training with a loss function in terms of error in microstructure estimation may lead to better generalizability to pathological brains than loss functions in terms of signal reconstruction error (Grussu et al., 2021). Other factors may also influence the generalizability of machine learning methods to pathological test data, but there has been little work to investigate this topic.

#### 5.1.2 Validation on external data

A number of studies have reported successful application of trained machine learning models on data from other scanners/centers without any data harmonization or retraining (Zheng et al., 2023). Other studies have suggested that when dMRI intensities or subject demographics deviate from training data, intensity normalization, data harmonization, or model retraining/fine-tuning may be necessary (Golkov et al., 2016;R. Lin et al., 2023). However, data harmonization in dMRI is a complex problem of its own as mentioned inSection 4.3. Moreover, there may be a tendency in the published studies not to report discouraging results when it comes to validation on external data. Given the significant diversity across datasets and applications and strong dependence of machine learning methods on training data distribution, model settings, and design choices, more extensive validations are needed to assess the true advantages of machine learning methods in real-world settings with heterogeneous data.

#### 5.1.3 Challenges of performing fair and extensive validations and comparisons

Studies often fail to perform careful and extensive comparison with competing methods. This makes it difficult to judge the merits of a new method and contrast its advantages and disadvantages compared with existing technology. This may be due to the inherent difficulties in performing such comparisons. For tractography, as an example,Poulin et al. (2019)have pointed out that factors such as dMRI preprocessing, streamline seeding approach, and training and test data distributions can be major confounding factors that make it difficult to compare machine learning techniques. Lack of standardized dMRI preprocessing pipelines has been a primary challenge for assessing nonmachine learning methods as well (Tax et al., 2022). Recent efforts to develop such pipelines (e.g.,Cai, Yang, Hansen, et al., 2021;Cieslak et al., 2021;Theaud et al., 2020) may greatly benefit validation and comparison of machine learning methods for dMRI analysis. Nonetheless, as new methods appear in the literature at an accelerated pace, progress in the field can only be achieved if new methods are properly compared with the state of the art.

### 5.2 Inherent limitations and drawbacks of machine learning approaches

Despite the indisputable advantages of machine learning methods, some of which have been discussed inSection 3, machine learning methods also suffer from inherent limitations. This section describes some of these, with a focus on applications in dMRI analysis.

#### 5.2.1 Dependence on the training data distribution

We pointed out that some machine learning models, such as neural networks, have a very high expressive capacity that allows them to learn complex functions from large training datasets. This property, however, can present itself as a downside. For example, the training data may not be sufficiently rich to enable learning of the true target function. In such a scenario, classical methods will likely continue to work as usual, whereas machine learning methods will probably make large and unpredictable errors (Gyori et al., 2022). In other words, machine learning methods can only be expected to work as far as the training data allows. For tractography, for instance, classical methods are likely to work well on brains with different morphology, whereas machine learning methods may fail if the test brain is vastly different from the training data.

Obtaining reliable training data is an important challenge in many dMRI applications discussed above. Many studies have resorted to using simulated data for model training. Realistic simulations, physical phantoms, and histologically validated dMRI data may be useful in many applications (Golkov et al., 2016). However, acquiring these types of data can often be very costly or infeasible.

#### 5.2.2 Unpredictable generalizability to unseen test data

Generalizability of machine learning methods to new test data can be affected by factors that may not be clear beforehand (Gyori et al., 2022). For instance, it has been suggested that factors such as signal intensity and acquisition parameters such as echo time may impact the performance of a neural network model to the extent that a complete retraining may become necessary (Golkov et al., 2016;Rensonnet et al., 2021). Performance of conventional (i.e., nonlearning) methods is probably much less affected by such unforeseen factors.

Closely related to the above point is the issue of out-of-distribution (OOD) data, which refer to data samples that are outside the distribution of the training data. Detecting OOD data in higher dimensional signal spaces is very challenging, and machine learning methods have no performance guarantee on OOD data (W. Liu et al., 2020;Ruff et al., 2021). A machine learning technique that works well on in-distribution data can experience unexpected and catastrophic failure on OOD test data. Obtaining training data that is sufficiently rich and heterogeneous to represent all data that will be potentially encountered at test time can be difficult if not impossible because many factors may influence the data distribution. In tractography, for example, data noise and artifacts can be important factors and the trained model may produce inferior results if the training and test data are different with regard to these aspects (Poulin et al., 2019;Théberge et al., 2021). Nonlearning methods are less prone to such failures.

It has been shown that machine learning methods can lead to systematic and unpredictable biases in microstructure mapping from dMRI signal (Gong et al., 2023;Gyori et al., 2022). Moreover, this bias can depend on factors such as SNR, partial volume effects, and distribution of the microstructure values in the training and test data (Gong et al., 2023;Gyori et al., 2022). As a result, predictions of these methods may lead to erroneous conclusions when studying the impact of pathology on microstructure (Gyori et al., 2022). While such biases may be present in many machine learning methods, discovering them requires extensive validation and analysis that is missing in most published studies. These suggest that machine learning methods may suffer from shortcomings that are not easy to recognize. In general, most modern machine learning methods are complex and difficult to interpret, a topic that is further discussed below inSection 5.5.

### 5.3 Modeling considerations

This section describes some of the technical considerations that commonly arise in developing machine learning models in dMRI.

#### 5.3.1 Data representation

The dMRI data used as input to machine learning models are typically in the so-called q-space, where each measurement in a voxel is associated with a gradient strength and a gradient direction. The gradient strength/direction is typically different between subjects/scans. Many different approaches have been used to uniformly represent the data.

- A simple approach is to interpolate the data unto a fixed spherical grid, where a typical grid size is approximately 100 (Neher et al., 2015).
- A common approach is representation in spherical harmonics. There exist several formulations for extending the spherical harmonics representation to multishell data (Cheng et al., 2011;Descoteaux et al., 2011).
- Zucchelli et al. (2021a,^2021^b) have advocated for signal representation in terms of their rotationally invariant features (Zucchelli et al., 2020). They have shown that, compared with the two common representations mentioned above, their representation leads to better estimation of multicompartment models (Zucchelli et al., 2021b) and FOD (Zucchelli et al., 2020).
- Another approach is sparse representation in dictionaries (Ye, Li, & Chen, 2019). InYe et al. (2020), dictionaries that were separable in spatial and angular domains (Schwab et al., 2016) were used. Compared with nonseparable dictionaries, separable formulations significantly reduce the number of parameters and may improve the analysis results (Ye et al., 2020).
- Graphs offer a flexible representation framework that has been successfully used by prior studies. InHong, Kim, et al. (2019), the authors constructed the adjacency matrix in terms of the difference between pairs of measurements. They computed the difference in terms of spatial distance, angular difference between gradient directions, and difference in gradient strengths. A similar representation was used byG. Chen et al. (2017)to extend the nonlocal means denoising algorithm to dMRI.
- Another approach, proposed byPark et al. (2021), consists of projecting the q-space data onto the standard (xy, xz, and yz) planes and binning them into fixed grids. For multishell data, each shell is binned separately.
- For applications that require large spatial fields of view, such as in tract segmentation (Wasserthal, Neher, & Maier-Hein, 2018), limited GPU memory typically does not allow for using the dMRI data as model input. In such applications, as mentioned above inSections 4.6,4.7, and4.9, DTI or FOD maps may be computed and used as input for machine learning models.

#### 5.3.2 Neural network architecture

Most deep learning studies discussed in this paper appear to have designed their network architecture in an ad-hoc way as they have provided little or no justification for their choices. One study (H. Chen et al., 2022) has reported successful application of network architecture search (Zoph & Le, 2016) for dMRI data prediction.

Standard convolutional operations are equivariant to translation, which is one of the key reasons for their success in computer vision. However, they are not equivariant to rotation. Rotation-invariance and rotation-equivariance (i.e., a rotation of the input to result in an identical rotation in the output) are desired in many applications. Incorporating these properties in the network architecture removes the need for the model to learn every possible orientation, which would otherwise require large datasets or massive data augmentation. It can improve the performance in various computer vision tasks (Esteves et al., 2018). In dMRI, the q-space data in each voxel can be represented as spherical maps. Therefore, in addition to the rotations in the voxel (physical) space, q-space rotations should also be accounted for (Elaldi et al., 2023). Most standard neural network models such as CNNs have been designed for signals in Euclidean space such as digital images. An ideal convolutional operation for q-space data should be either rotation-invariant (e.g., for estimating scalar maps of tissue microstructure) or rotation-equivariant (e.g., for FOD estimation). Moreover, it should allow for irregular sampling on the sphere.

Müller et al. (2021)have argued that standard (i.e., nonrotationally equivariant) networks, when applied in dMRI, are at the risk of both underfitting and overfitting. This is because they need to learn the rotation-equivariance relationships from training data, which may be biased toward certain orientations. Incorporating rotation-equivariance will eliminate the need for learning these constraints, enable better weight sharing, improve generalization to orientations not seen in the training data, and allow the model to concentrate on learning the actual underlying function of interest (Elaldi et al., 2021;Müller et al., 2021).

Various techniques have been proposed to build rotation-equivariance into neural networks and to extend standard convolutions to signals on the sphere. Some of these designs are described below.

- Some studies have proposed to discretize the surface of the sphere and to apply standard 2D convolutions on the resulting grid (Boomsma & Frellsen, 2017;Coors et al., 2018;Su & Grauman, 2017). A straightforward discretization can use an equiangular spacing in the standard spherical-polar coordinate system.Boomsma and Frellsen (2017)suggest that a better discretization can be achieved by using the “cubed sphere” representation originally developed byRonchi et al. (1996). In this representation, each point on the sphere is represented by the coordinates in one of the six patches of a circumscribed cube. They define cubed-sphere convolution as applying a standard 2D convolution on a uniformly spaced grid on the six sides of the cube. This method can be extended to concentric shells.
- A common approach in computer vision is to divide the surface of the sphere into small regions and process each region as a planar signal with standard 2D convolutions (Fluri et al., 2018;Gillet et al., 2019). This processing is usually followed by hierarchical pooling. However, this approach does not result in rotation-equivariance.
- Another approach is based on representing the data as a graph.Perraudin et al. (2019)proposed DeepShere, which processes spherical signals by representing the sphere as a graph. DeepShere relies on filters that are restricted to being radial. As a result, the convolutional operations are rotation-equivariant and the network predictions become invariant or equivariant to input rotations. Moreover, this formulation has a lower computational cost than performing the convolution with spherical harmonics.
- Various other techniques have been used to develop rotation-equivariant convolutional operations. The study ofT. S. Cohen et al. (2018)is based on the spherical Fourier transform, where the convolution is performed in the spectral domain based on the convolution theorem.Banerjee et al. (2020)have developed a method based on Volterra’s function.Bouza et al. (2021)have proposed a manifold-valued convolution based on a generalization of the Volterra Series to manifold-valued functions, whereasEsteves et al. (2018)propose a rotation-equivariant 3D convolutional network based on spherical harmonics.T. Cohen and Welling (2016)have proposed group equivariant convolutional networks. Group convolutions enable equivariance with regard to rotation, thereby increasing the expressive power of convolutions without increasing the number of parameters.
- Müller et al. (2021)have generalized rotation-equivariant neural networks to the 6D space of dMRI data. They have developed a new linear layer that is equivariant under rotations in the voxel space and q-space and under translations in the voxel space. Their formulation enables translation-equivariance in the coordinate space and rotation-invariance in the coordinate space and q-space.

Numerous studies have reported that these specialized network architectures can improve the performance of deep learning models in different dMRI analysis tasks (Bouza et al., 2021;Goodwin-Allcock et al., 2022;Kerkelä, Seunarine, Szczepankiewicz, & Clark, 2022;Koppers & Merhof, 2021;R. Liu, Lauze, Bekkers, et al., 2022).Bouza et al. (2021)found that their proposed convolution operation improved the state of the art in classification of Parkinson’s disease patients versus healthy controls and in FOD reconstruction.Sedlar et al. (2021)reported improved estimation of NODDI parameters with a rotationally invariant CNN, whileR. Liu, Lauze, Erleben, et al. (2022)showed that their network resulted in a 10% reduction in the number of trainable weights without a reduction in performance in a tissue segmentation application.Kerkelä, Seunarine, Szczepankiewicz, and Clark (2022)found that a rotation invariant spherical CNN performed better than a standard MLP in estimating a multicompartment model. For angular super-resolution of dMRI data,Lyon et al. (2023)showed that a parametric continuous convolution network outperformed a standard CNN while reducing the number of parameters by an order of magnitude. Other dMRI studies have shown that rotationally equivariant networks can lead to more accurate tissue segmentation and lesion segmentation compared with standard networks (R. Liu, Lauze, Bekkers, et al., 2022;Müller et al., 2021).Goodwin-Allcock et al. (2022)have shown that including rotation equivariance improves the generalizability of deep learning models to new gradient schemes and reduces the required training data size.

#### 5.3.3 Optimization objective functions

For tissue microstructure mapping, one study compared a training loss function based on the error in microstructure prediction with another in terms of the error in signal prediction (Grussu et al., 2021). The former has been by far more common in machine learning methods, whereas the latter is the common loss function in standard optimization-based methods. It was shown that although the former led to lower microstructure prediction errors when the noise was modeled accurately, the latter resulted in lower signal reconstruction error and it was more practical because it eliminated the need for reference microstructure and enabled training with synthetic and real data (Grussu et al., 2021). The authors speculated that the weakness of a loss function based on signal reconstruction error could be due to the fact that neural networks might tend to learn features that originate from the noise floor. In the context of IVIM model fitting, a similar method of training based on the signal prediction error was proposed byKaandorp et al. (2021), where the authors used the terms “unsupervised” and “physics-informed” to describe their method.Parker et al. (2023)argue that the use of root mean square loss function is not consistent with the measurement noise distribution in dMRI. They propose a loss based on Rician likelihood and show that, for estimation of apparent diffusion coefficient (ADC) and IVIM parameters, it leads to lower estimation bias at lower SNR.

Another study on tissue microstructure estimation has argued that loss functions based purely on the predicted signal are inadequate (G. Chen et al., 2023). Instead, the authors propose two alternative loss functions that additionally incorporate the error in microstructure estimation. One of their loss functions consists of the$\ell_{1}$norms of the errors of the dMRI signal and the microstructure indices. The other loss function, which they name the spherical variance loss, is based on the spherical variance of the dMRI signal that is correlated with microstructure (Huynh et al., 2019). Experiments show that, compared with standard loss functions such as the$\ell_{1}$,$\ell_{2}$, and Huber loss of the signal prediction error, these two augmented loss functions result in more accurate prediction for a range of parameters, including GFA, DKI, and NODDI. However, they do not compare these new loss functions with a loss function based on the error in the predicted tissue microstructure index.

### 5.4 Data, ground truth, and evaluation

For many dMRI applications, obtaining data with reliable target/label is difficult or impossible.

#### 5.4.1 Synthesizing data from high-quality dMRI scans

Most commonly, high-quality dMRI measurements have been used to generate the data needed for developing and validating machine learning methods. For tract segmentation/registration,B. Li, Niessen, Klein, de Groot, et al. (2021)used an automatic method based on probabilistic tractography and atlas-based segmentation to generate their training labels. For tissue microstructure mapping and for FOD estimation, most studies have used dense q-space data and estimated ground truth parameters using standard methods (Koppers, Friedrichs, & Merhof, 2017;Koppers, Haarburger, Edgar, et al., 2017;Nath et al., 2020;Yao, Newlin, et al., 2023;Zheng et al., 2023).

#### 5.4.2 Histology/microscopy

In certain applications, histological data can be used to establish a reasonable ground truth, but at considerable cost. For estimation of intra-axonal water exchange time,Hill et al. (2021)used histology analysis of an in vivo mouse model of demyelination using electron microscopy to generate ground truth. Specifically, they measured the thickness of the myelin sheath as a measure of exchange time. Surprisingly, they found that the prediction of their random forest regression model based on dMRI signal was strongly correlated with histological measurements, with a Pearson correlation coefficient of 0.98.Nath, Schilling, et al. (2019)used confocal histology to generate data for FOD estimation. Since the data obtained with this technique were costly and very limited, they performed massive data augmentation to increase the effective size of their data. For data harmonization, a large purely dMRI scan–rescan dataset was used to boost a small dataset of paired histology and dMRI (Nath, Parvathaneni, et al., 2019). Another study used coregistered dMRI and histology data to develop a machine learning method for estimating several microstructural indices and achieved higher estimation accuracy than standard methods (Fick et al., 2017). For validating dMRI-based tract segmentation methods,Lucena et al. (2022,^2023^) have advocated for using navigated transcranial magnetic stimulation to establish a ground truth.

#### 5.4.3 Annotations from human experts

In some applications, such as those involving segmenting or analyzing specific white matter tracts, labels can be obtained from human experts. This approach, however, may suffer from difficulty of defining anatomical ground truth (Wasserthal, Neher, & Maier-Hein, 2018), high intra- and interexpert variability (K. G. Schilling et al., 2021a;Wakana et al., 2007), and labeling errors. Such label errors need to be properly accounted for using advanced machine learning techniques (Karimi, Dou, et al., 2020). In applications such as tractography and tract analysis, obtaining labels from multiple experts may be useful but costly (Poulin et al., 2019;Théberge et al., 2021;Yendiki et al., 2011).

#### 5.4.4 Data simulation

Another approach is to use simulation techniques such as the Monte Carlo method. This approach has been widely used for tissue microstructure mapping (Grussu et al., 2021;Gyori et al., 2022;Hill et al., 2021). Despite its limitations, data simulation is sometimes the only reasonable approach when high-quality data are not available to compute a reliable ground truth. For microstructure estimation, numerical simulations and physical phantoms are considered valid and powerful methods for developing and validating computational methods (Fieremans & Lee, 2018;Jelescu et al., 2020). Data simulation has two potential advantages: (i) it enables exploring the full range of parameters that may influence the signal and (ii) the ground truth target is known accurately (Gyori et al., 2022;Masutani, 2019). Because of the flexibility of data simulation methods, they may also be useful for investigating what data acquisition protocols lead to more accurate reconstruction. This approach was used byLee et al. (2021)to determine the optimal b values for IVIM reconstruction with deep learning.Gyori et al. (2022)investigated the impact of training data distribution on the test performance of supervised machine learning methods for microstructure mapping. When training on simulated data, they found that in order for the model to achieve reasonable accuracy on atypical brain tissue, the training data should be synthesized using the complete range of plausible parameter values. When the parameter space used to generate the training data matched that of healthy brains, estimation accuracy on atypical brains was low, although the estimations showed a deceptively high precision.

There have been innovative approaches to dMRI data simulation in prior studies.Nedjati-Gilani et al. (2017)used Monte Carlo simulation to synthesize dMRI data based on histologically valid microstructure parameters.Ye, Cui, and Li (2019)have developed a method for synthesizing q-space data that can potentially be used for any machine learning application in dMRI. Their signal generator is an MLP that is trained to minimize the difference between the distribution of the synthesized signal and that of the observed signal. They use a continuous representation of the dMRI data in the SHORE basis (Ozarslan et al., 2009). Their preliminary experiments have shown promising results for estimation of the NODDI model.

Qin et al. (2020)have proposed and validated a knowledge transfer method that relies on a source domain where high-quality data are available. Using the SHORE basis, this method interpolates the source data in the q-space and/or voxel space to synthesize data that match the quality of the target data. Model training is carried out in the source domain and transferred to the target domain. For Gibbs artifact suppression, one study trained a CNN on more than one million natural images and simulated more than 10 million data instances (Muckley et al., 2021). Experiments showed that the size and richness of the collection of natural images used for training were essential to ensuring generalizability of the model to dMRI test data.Graham et al. (2016)developed a simulation method to enable direct quantitative evaluation of artifact-correction methods. They subsequently used this method to develop and validate a deep learning motion artifact detection technique (Graham et al., 2018). Their study showed that with proper data simulation, only a small real dataset was sufficient to calibrate the method.

Using simulated data is typically much more convenient than using real-world data. Some studies have relied solely on simulated data to train and/or evaluate their methods (Gong et al., 2023;Kerkelä, Seunarine, Henriques, et al., 2022;Parker et al., 2023;Weine et al., 2022). However, using in silico simulations to synthesize training data may be inadequate because some factors such as the noise distribution are difficult to model accurately (Grussu et al., 2021). For IVIM-DKI estimation, one study observed that increasing the noise level in the synthesized training data improved the model’s test accuracy (Bertleff et al., 2017).Masutani (2019)analyzed the impact of matching the noise level between training and test data. He found that the best test performance was achieved when the noise level in the training data was close to or slightly higher than the noise level in the test data.de Almeida Martins, Nilsson, Lampinen, Palombo, While, et al. (2021), in contrast, found that training with a much noisier dataset could still result in superior results on test data with relatively lower noise. For DKI estimation, another study found that varying the noise level in the synthetic training data led to better results on clinical test scans compared with a model that was trained using a single noise level (Masutani et al., 2021). In some applications, such as cardiac DTI (Weine et al., 2022) and fetal imaging (Karimi, Jaimes, et al., 2021) mentioned above, obtaining reliable in vivo data faces particular difficulties such that using synthetic training data may be unavoidable.

Another important consideration is the choice of evaluation metrics. It is often unclear what metrics should be reported for a comprehensive assessment of a new method and a fair comparison with competing techniques. As an example, for tissue microstructure mapping, most studies report the RMSE as the main or the only metric. However, it has been suggested that correlation analysis and sensitivity analysis are important for revealing certain limitations of machine learning methods that cannot be assessed based solely on RMSE (de Almeida Martins, Nilsson, Lampinen, Palombo, Westin, & Szczepankiewicz, 2021). Moreover, rather than reporting a single global RMSE value for a method, detailed analysis of the estimation error such as its variation in different brain regions may reveal biases that are not captured with global RMSE (Epstein et al., 2022;Gong et al., 2023;Gyori et al., 2022;Karimi, Warfield, et al., 2021).

Although for many dMRI computations obtaining the ground truth may be difficult or impossible, test–retest reproducibility assessments may be far easier to perform. In fact, repeatability tests have often been used to assess machine learning methods in dMRI. For example,Tunç et al. (2014)used repeated scans of the same subjects to assess the repeatability of their tract segmentation method, whileBarbieri et al. (2020)computed intersubject variability to validate a deep learning method for IVIM estimation. Performing such tests has become more feasible with the growing availability of public datasets that include repeated scans of the same subjects on the same scanner (Chamberland et al., 2019) or on different scanners (Cai et al., 2021b) or on different scanners with different protocols (Cai et al., 2021a;Ning et al., 2020).

Where an objective/quantitative ground truth is difficult to establish, visual assessment by human experts may be a viable alternative. One study quantified the agreement between two readers in their interpretation of IVIM parameter maps to compare a deep learning method with conventional estimation methods (Barbieri et al., 2020). However, the authors have pointed out that this approach may favor methods that wrongly compute consistent results while failing to account for genuine heterogeneity. Another study has reported successful validation of a deep learning method for fetal DTI estimation based on blind randomized assessment and scoring by neuroanatomists and neuroradiologists (Karimi, Jaimes, et al., 2021).

Lack of reliable and standardized data for developing and validating machine learning methods may also be one of the main barriers to quick and widespread adoption of these methods. Despite repeated claims that machine learning methods can outperform classical techniques, these methods have not seen widespread adoption. For tractography, for example,Poulin et al. (2019)point out that despite repeated demonstration of the capabilities of machine learning methods, none of these methods have been adopted for real-world applications. They attribute this, in part, to the lack of well-defined test benchmarks to allow conclusive performance evaluation and comparison.

An effective approach to assessing the potential of machine learning for dMRI analysis is via open competitions, where research teams are free to develop and apply standard nonlearning methods and machine learning techniques. A good example is a recent open challenge where research teams were invited to develop deep learning methods for estimating diffusion tensor parameters from reduced measurements (Aja-Fernández et al., 2023). Specifically, focusing on distinguishing between episodic and chronic migraine patients, the goal was to investigate whether deep learning methods were able to achieve the same level of sensitivity and specificity when using 21 measurements, compared with 61 measurements typically used with standard methods for this analysis. With conventional estimation methods, 60% of the differences detected by TBSS with 61 measurements are missed when using 21 measurements. A total of 14 research teams participated in the challenge. Results showed that deep learning methods improved the global image metrics such as PSNR and SSIM. They also improved the sensitivity of TBSS. However, these improvements came at the cost of higher false positive rates, which increased linearly with the true positive rate. The teams had used different deep learning models and had adopted various approaches, such as mapping the reduced measurements directly to the target DTI parameters or estimating dense dMRI measurements first. The study concluded that the results of deep learning methods should be interpreted with caution even when global image quality metrics are improved.

A very appealing approach to assessing the new methods is to use measures of patient outcome as the evaluation target. This approach has been successfully attempted in a few studies, where the predicted microstructure indices have been correlated with poststroke outcome (Gibbons et al., 2019) and outcome of pancreatic cancer patients (Kaandorp et al., 2021). Another study used acute-phase dMRI (as well as anatomical MRI) data as input to a small neural network to directly predict the chronic size of ischemic lesions, where the T2-weighted images at 3 months after stroke were used to establish the ground truth (Bagher-Ebadian et al., 2011).

### 5.5 Explainability, uncertainty, and reliability

Advanced machine learning methods such as deep neural networks are increasingly used in safety-critical applications such as medicine. However, they are very complex and hard to interpret (Arnez et al., 2020;Arrieta et al., 2020). It is well documented that deep learning models produce overconfident and poorly calibrated predictions (C. Guo et al., 2017;Lakshminarayanan et al., 2017a), they can be fooled into making erroneous predictions (I. J. Goodfellow et al., 2014;Kurakin et al., 2016), and they fail silently on OOD data (Bulusu et al., 2020;Mårtensson et al., 2020). There has been much effort to address these issues (Bulusu et al., 2020;Loquercio et al., 2020). The majority of these efforts have focused on classification problems. Deep learning-based regression has received much less attention. For example, while for classification there is a widely accepted definition of uncertainty calibration (C. Guo et al., 2017;Lakshminarayanan et al., 2017a), for regression there is much disagreement (Kuleshov et al., 2018;Levi et al., 2019).

Even though very few studies have investigated these issues for machine learning-based dMRI analysis, they have reported important observations. For example, it has been reported that machine learning methods produce deceptively confident (i.e., high-precision) predictions on noisy test data, while with classical estimation methods, higher measurement noise levels are properly reflected in predictions as higher variance (Gyori et al., 2022). Unfortunately, very little work has been devoted to characterize these issues and to devise effective solutions.

A few studies have incorporated uncertainty estimation in their methods. For microstructure mapping,Ye et al. (2020)quantified the uncertainty of their neural network-based tissue microstructure estimation using a residual bootstrap technique. They validated their uncertainty computation method by assessing the correlation with estimation error and inspecting the confidence intervals. They concluded that this method could compute practically useful uncertainty estimates. For diffusion tensor estimation, another study computed data-dependent uncertainty via loss attenuation and model uncertainty via Monte Carlo dropout (Karimi, Warfield, et al., 2021). Their extensive experiments showed that estimation uncertainties computed by their proposed methods could highlight the model’s biases, detect domain shift, and reflect the measurement noise level.Avci et al. (2021)have shown that a simple uncertainty quantification method based on dropout reduces the prediction error and can also serve as an indicator of pathology or measurement artifacts. For IVIM estimation, one study estimated the full Gaussian posteriors (i.e., mean and variance) for the parameters and used the standard deviation of the posterior as a proxy for estimation uncertainty (L. Zhang et al., 2019). Experiments with simulated and real data showed that the computed uncertainties were qualitatively meaningful. For tract segmentation, one study has used dropout and test–time augmentation to compute epistemic and aleatoric uncertainties (Lucena et al., 2022). After applying post hoc uncertainty calibration, the computed uncertainty is shown to be well correlated with segmentation error. The authors argue that such uncertainty computations can be useful in surgical planning applications such as for reliable delineation of eloquent areas. In general, it may be easier to probe the explainability of conventional machine learning models than large neural networks. For example, for microstructure estimation, one study was able to extensively examine the importance of different feature sets and different measurement shells in the q-space for a random forest model (Fick et al., 2017). Performing a similar analysis for a deep learning model should be more challenging due to the longer training times for these models.

In the context of dMRI super-resolution,Tanno et al. (2017,^2019^) estimated the uncertainty for CNN and random forest models. For their CNN estimator, they employed a heteroscedastic noise model to compute the intrinsic uncertainty and variational dropout to compute the model uncertainty (Tanno et al., 2017). However, their evaluation of estimation uncertainty relied on a qualitative visual inspection of the correlation between uncertainty and estimation error. They showed that the uncertainty was higher for brain pathologies not observed in the training data and that the computed estimation uncertainty could be useful for downstream computations such as tractography. In another study (Tanno et al., 2019), they employed similar techniques to compute the estimation uncertainty for dMRI super-resolution based on the IQT approach discussed above. They also developed methods to propagate the uncertainty estimates to downstream analyses such as computation of microstructure indices. Experiments showed that uncertainty quantification could highlight model’s failures, explain prediction performance, and improve prediction accuracy. Also in the context of super-resolution,Qin, Liu, et al. (2021)used a method based on model ensembles (Lakshminarayanan et al., 2017a) to quantify the uncertainty for estimation of NODDI parameters. They showed that the computed uncertainties had moderate correlation with estimation error and could be used to improve estimation accuracy.

Model interpretability has also received little attention.H. Xu et al. (2019)used attention maps (similar toK. Xu et al., 2015) to interpret the decision mechanism of a tractography-based tract segmentation model. They showed that the attention maps provided useful insights that were consistent with the anatomy of the fiber tracts connecting different brain regions. For subject-level classification based on tract-specific tissue microstructure, one study has shown that class activation maps can identify the white matter tracts that underlie the group differences (F. Zhang, Xue, et al., 2022).Varadarajan and Haldar (2018)criticized nonlinear machine learning FOD estimation methods for their black-box nature and difficulty of predicting their performance on unseen measurement schemes. They formulated FOD computation as a linear estimation problem and achieved prediction accuracy on par with or better than standard methods. One can argue that studies ofYe (2017c)andYe, Li, and Chen (2019)that involve unfolding the iterative algorithms for microstructure estimation also represent a step toward improving the explainability of deep learning models.Rensonnet et al. (2021)have further improved Ye’s study in this direction by proposing a hybrid method that involves the use of a physics-based fingerprinting dictionary within a deep learning framework. Their method first projects the dMRI signal into a dictionary containing physics-based responses. Subsequently, an MLP uses the sparse representations computed in the first step to compute the target microstructure indices. Compared with an MLP that is trained end-to-end, this new method showed competitive accuracy while having qualitatively more interpretable features.

Better model explainability may also aid in designing more effective computational methods. For example, it is unclear why machine learning methods often achieve more accurate tissue microstructure estimation than standard optimization-based methods. The shortcomings of optimization methods are due to several factors including sensitivity to measurement noise, poor initialization, and degeneracy (i.e., presence of multiple equally optimal solutions). For DTI estimation, which consists in a linear or nonlinear least squares problem, the advantage of deep learning models is likely only due to their ability to use the measurements in neighboring voxels to reduce the impact of measurement noise. For multicompartment models with a complex optimization landscape, in contrast, machine learning models may actually be able to learn to avoid local minima and compute better estimates.

## 6 Conclusions

Machine learning has the potential to usher in a new generation of computational techniques for dMRI data processing and analysis. Some of the capabilities that have been demonstrated by multiple studies include denoising, artifact correction, data harmonization, estimation of microstructural indices, tractography, and tract analysis tasks such as segmentation and registration. Compared with conventional computational techniques in dMRI, the new machine learning methods may offer improved performance in several aspects including faster computation, ability to handle imperfect data, flexible modular design, end-to-end optimization, seamless and easy integration of spatial information, and other sources of information such as anatomical MRI. However, in order to realize the full potential of these methods, we need to overcome several critical limitations and remaining issues. More rigorous validation on rich and heterogeneous datasets is necessary. Standardized data preprocessing pipelines, validation benchmarks, and evaluation metrics can dramatically help the research community in developing and assessing more effective methods and discovering truly meritorious techniques. Finally, enabling model explainability and proper uncertainty estimation may facilitate and expedite the adoption of these methods in clinical and scientific applications.

## Data Availability

This paper does not include any data or code.
